# Tumor-derived exosomal miR-199b-5p promotes proliferation and epithelial-mesenchymal transition in non-small cell lung cancer by targeting CCNL1

**DOI:** 10.1016/j.tranon.2025.102564

**Published:** 2025-10-16

**Authors:** Bangzhu Liu, Yan Rui, Miao Li, Linian Huang

**Affiliations:** a Department of Respiration and Critical Care Medicine, Wuhu Hospital, East China Normal University (The Second People’s Hospital of Wuhu City), Wuhu, Anhui 241000, China; b Department of Respiration and Critical Care Medicine, The First Affiliated Hospital of Bengbu Medical University, Bengbu, Anhui 233000, China; c Department of General Medicine, The First Affiliated Hospital of Bengbu Medical University, Bengbu, Anhui 233000, China

**Keywords:** Exosomes, Epithelial-mesenchymal transition, Cell cycle, Wnt/β-catenin

## Abstract

**Objective:**

To explore the effect of exosome-mediated miR-199b-5p on lung cancer cells behavior, intrapulmonary metastasis, and its underlying mechanism.

**Methods:**

Exosomes from SK-LU-1 cells overexpressing has-miR-199b-5p (miR-199b-5p) were used to treat A549 or H299 cells. Cell motility was evaluated using wound scratch healing and transwell assays. Gene and protein expression were detected by quantitative real-time PCR (QRT-PCR) and Western blot. Target genes of miR-199b-5p were predicted through multiple-database analysis and validated.

**Results:**

Transmission electron microscopy (TEM) demonstrated isolated exosomes had a typical bilayer membrane (30-100 nm). miR-199b-5p was highly expressed in lung cancer cells and detectable in the serum of lung cancer patients, with the highest levels observed in SK-LU-1 cells and their derived exosomes. Exosomal miR-199b-5p significantly enhanced the motility of A549 and H1299 cells and upregulated the expression of epithelial-mesenchymal transition (EMT)-related proteins. Exosomes promoted lung metastasis of H1299 cells and inhibited the expression of Cyclin L1 (CCNL1). Either CCNL1 inhibition or miR-199b-5p overexpression significantly promoted H1299 cell proliferation and reduced apoptosis, while CCNL1 overexpression inhibited cell motility. Protein-protein interaction (PPI) network, Gene Ontology (GO), and Kyoto Encyclopedia of Genes and Genomes (KEGG) pathway analyses indicated involvement of the Wnt/β-catenin signaling pathway in the downstream mechanism. Mechanistically, miR-199b-5p inhibited cyclin-dependent kinase 11 (CDK11) and upregulated the expression of transcription factor 4 (TCF-4) and β-catenin.

**Conclusions:**

Overexpression of miR-199b-5p promotes proliferation, migration, and EMT in lung cancer cells by targeting CCNL1, with its downstream regulatory effects mediated through the Wnt/β-catenin signaling.

## Highlights

• MiR-199b-5p showed the highest expression level in SK-LU-1 cells and its exosomes.
• Overexpression of miR-199b-5p promotes lung cancer cell proliferation, migration, and EMT.
• The underlying mechanism of miR-199b-5p was related to CCNL1.

## Introduction

Lung cancer is characterized by a high incidence and mortality rate, with patients exhibiting low survival rate and poor prognosis. Non-small cell lung cancer (NSCLC) accounts for approximately 80 % of all lung cancer cases [1]. A large proportion of NSCLC patients are diagnosed at advanced stages, frequently accompanied by extensive metastasis, which significantly contributes to their unfavorable clinical outcomes [2]. Despite substantial advancements in lung cancer diagnosis and treatment in recent years, patient prognoses remain suboptimal. This persistent challenge is primarily attributed to late-stage diagnosis and the limited efficacy of current therapeutic strategies [3,4]. Therefore, the identification of novel biomarkers for early detection, prognostic assessment, and evaluation of treatment response in lung cancer is of critical importance.

MicroRNAs (miRNAs) are a class of short non coding RNAs that post-transcriptionally regulate target gene expression through complementary pairing with the 3′- UTR ends of mRNAs [5]. Accumulating evidence has demonstrated that numerous miRNAs influence tumor growth, immune evasion, and cisplatin resistance in NSCLC [[6], [7], [8], [9]]. The miRNA-199 family comprises two highly conserved subtypes: miRNA-199a and miRNA-199b, among which miR-199b-5p exhibits significant tumor- suppressor potential [10]. Previous studies have linked miR-199b-5p expression to metastatic progression in various tumors, including medulloblastoma, gastric cancer, endometrial cancer, and breast cancer [[11], [12], [13], [14]]. Peng et al. reported that LINC81507, a long non-coding RNA downregulated in NSCLC, acts as a molecular sponge for miR-199b-5p [15]. However, the specific role of miR-199b-5p in NSCLC remains unclear.

Extracellular vesicles (EVs) are key mediators of intercellular communication, distinguished by their lipid bilayer structures [16]. Exosomes, a major subtype of EVs, encapsulate diverse bioactive molecules such as miRNAs, mRNA, and proteins [17]. Upon uptake by recipient cells, these cargo molecules transmit signals from donor cells, thereby regulating biological processes including cell proliferation, migration, and invasion. Notably, malignant cells secrete higher quantities of EVs compared to normal cells, and these EVs can be isolated from bodily fluids. Pathological conditions, particularly cancer, can alter the biogenesis and content of EVs [18]. Exosomal miRNAs have emerged as promising candidates for tumor diagnosis and treatment, as various exosomal miRNAs have been shown to regulate the initiation and progression of NSCLC.

In this study, we isolated exosomes from both healthy volunteers and lung cancer patients to detect miR-199b-5p We then transfected SK-LU-1 cells with a miR-199b-5p mimic, isolated exosomes from these transfected cells, and incubated them with A549 or H1299 cells. The functional impact of miR-199b-5p on NSCLC was evaluated through a combination of *in vitro* and *in vivo* experiments. Our study identify a novel potential therapeutic target for NSCLC treatment.

## Material and methods

### Exosome isolation from serum samples and cell culture supernatants

Healthy volunteers (*n* = 30) and NSCLC patients (*n* = 30) from Wuhu Second People's Hospital between March 2021 and October 2021. Exosomes were isolated from serum using an exosome separation reagent (Qiangen, Beijing, China) following the manufacturer’s protocol. Briefly, blood samples were centrifuged at 1000 × *g* for 10 min at 4 °C to remove cellular debris, the supernatant was further centrifuged at 10,000 × *g* for 10 min at 4 °C to eliminate residual cellular components. The morphology of the exosomes was observed using transmission electron microscopy (TEM), and the presence of exosomes was confirmed by the markers TSG101 and CD81. Exosome size distribution and particle concentration were analyzed using a nanoparticle analyzer (N30E, NanoFCM, Xiamen, China). Detailed protocols are provided in the supplementary material. Inclusion and exclusion criteria: NSCLC patients, included with a clear pathological diagnosis of NSCLC, excluded if diagnosed with other tumor types or mixed types. For healthy controls, included with no history of tumors or severe diseases, and age-matched to the patient group; excluded if taking medications that may affect serum exosome levels. This study was approved by the Medical Ethics Review of The Second People's Hospital of Wuhu City (Approve number: 2022 [8]).

Cell culture supernatants were collected and centrifuged at 3000 × *g* for 10 min to remove cellular debris. The supernatant was then filtered through a 0.22 μm membrane to eliminate large vesicles. Exosomes were pelleted by ultracentrifugation at 100,000 × *g* for 60 min at 4 °C. The resulting white precipitate was resuspended in 10 mL of sterile phosphate-buffered saline (PBS) and subjected to a second ultracentrifugation at 100,000 × *g* for 60 min to wash away contaminants. Purified exosome pellets were collected, and their morphology was verified by TEM.

### Cell culture

The human normal bronchial epithelial cell line BEAS-2B and human lung cancer cell lines (H1299, A549, and SK-LU-1) were obtained from the Cell Bank of the Chinese Academy of Sciences (Shanghai, China). BEAS-2B cells were cultured in Dulbecco's Modified Eagle Medium (DMEM, Hyclone, USA) supplemented with 10 % fetal bovine serum (FBS, Thermo Fisher Scientific, USA) and 1 % penicillin-streptomycin. SK-LU-1 cells were maintained in Minimum Essential Medium (MEM) from Hyclone, USA, H1299 and A549 cells were cultured in RPMI 1640 medium (Hyclone, USA).

### Cell transfection

Cells were seeded in 6-well plates and transfected when reaching 60–70 % confluence, following the protocol provided with the RNA transfection kit (BIOMEDICAL, China). For miRNA/siRNA transfection: 3 μg of miR-199b-5p mimic, negative control mimic (miR-NC), CCNL1 siRNA, or siRNA negative control (si-NC) was diluted in 100 μL of serum-free DMEM, then mixed with 5 μL of transfection reagent and incubated at room temperature for 15 min. The transfection mixture was added to the 6-well plate, and after 8 h of incubation, the medium was replaced with fresh complete medium. Cells were harvested for subsequent experiments at 24 or 48 h post-transfection. For CCNL1 overexpression, cells were transfected with a CCNL1 overexpression plasmid (pCDH—CMV-MCS-EF1-CopGFP-T2A-Puro) or empty control vector (NC), both constructed by Tsingke Biotech (Beijing, China), using the same transfection protocol.

### Wound scratch healing assay

Cells were seeded in 6-well plates and cultured until reaching 80 % confluence. A straight scratch was created in the monolayer using a 200 μL pipette tip. Detached cells were removed by gently washing the wells twice with serum-free medium, and fresh medium was added. Cell migration was monitored and photographed at 0, 24, and 48 h using a light microscope at 200× magnification. The scratch closure rate was calculated to quantify migratory capacity.

### Transwell assay for migration and invasion

For the invasion and migration assays, cells (1 × 10^4 per well) were resuspended in serum-free medium and seeded into the upper chamber, with Matrigel (BD, USA) applied for invasion assays and omitted for migration assays. The lower chamber was filled with 600 μL of complete medium containing 10 % FBS. After 36 h of incubation, non-migrated/non-invaded cells in the upper chamber were removed with a cotton swab. Cells that had migrated or invaded through the membrane were fixed with 4 % paraformaldehyde for 15 min, stained with 0.1 % crystal violet (Sigma-Aldrich, USA) for 20 min, and rinsed with PBS. Stained cells were counted and imaged using a microscope (Mshot, Guangzhou, China) at 200× magnification.

### Luciferase reporter assay

Human embryonic kidney 293T cells were seeded in 24-well plates and cultured for 24 h. To verify the interaction between miR-199b-5p and CCNL1, cells were co-transfection with miR-199b-5p mimics and CCNL1 3′-untranslated region (3′-UTR) luciferase reporter vectors (wild-type or mutant). A dual luciferase reporter assay system (Beyotime, Shanghai, China) was used to measure luciferase activity 24 h post-transfection. In brief, cells were lysed with 200 μL of lysis buffer per well, and 20 μL of cell lysate was mixed with 100 μL of firefly luciferase assay reagent to determine firefly luciferase activity using a luminometer. Subsequently, 100 μL of Renilla luciferase assay reagent was added to the same reaction to measure Renilla luciferase activity. Relative luciferase activity was calculated by normalizing firefly luciferase activity to Renilla luciferase activity (internal control) and expressed as arbitrary units. All experiments were conducted in triplicate [19].

### Quantitative real-time PCR (QRT-PCR)

AG RNAex Pro RNA (Accurate Biology, Hunan, China) was used to extract total RNA from cells and extracellular vesicles in each group, according to PrimeScript ™ RT reagent Kit (Takara, Japan) instructions for synthesizing cDNA, according to SYBR Prime Script RT-PCR Kit (Takara, Japan) was used for qRT-PCR reaction, with a reaction program of 95 °C for 3 min and one cycle; 95 °C for 15 s, 60 °C for 1 min, 40 cycles. Using U6 as the internal reference, 2^−ΔΔCt^ methods was used to calculate the relative expression level of miR-199b-5p. The primers were listed as follows, miR-199b-5p, forward, 5′- GCGCCCAGTGTTTAGACTATCTG-3′, reverse, 5′-ATCCAGTGCAGGGTCCGAGG-3′, U6: forward, 5′-GGAACGATACAGAGAAGATTAGC-3′, reverse, 5′-TGGAACGCTTCACGAATTTGCG-3′; CCNL1, forward, 5′-TACCATCGACCACTCTCTGATT-3′, reverse, 5′-GGATGCGTAAGTCCGTCTCAC-3′; GAPDH, forward, 5′-GGAGCGAGATCCCTCCAAAAT-3′, reverse, 5′-GGCTGTTGTCATACTTCTCATGG-3′.

### Western blot

Total protein was extracted following the protocol provided with the protein lysis solution (Solarbio, Beijing, China). Protein concentration was quantified using a BCA assay kit, after which sodium dodecyl sulfate-polyacrylamide gel electrophoresis (SDS-PAGE) was performed. Electrophoresis was initially conducted at 80 V for 40 min; once the proteins entered the separation gel, the voltage was increased to 120 V for an additional 90 min to achieve adequate separation. Subsequently, proteins were transferred to a polyvinylidene fluoride (PVDF) membrane (Millipore, USA) at a constant current of 300 mA for 90 min. The PVDF membrane was incubated overnight at 4 °C with the following primary antibodies: anti-CCNL1 (1:1000, cat. no. 13138–1-AP, Proteintech, Wuhan, China), anti-ARHGAP21 (1:1000, cat. no. PA5–100388, Thermo Fisher Scientific, USA), anti-EIF5B (1:1000, cat. no. 13527–1-AP, Proteintech, Wuhan, China), anti-TCF4 (1:1000, cat. no. 22337–1-AP, Proteintech, Wuhan, China), anti-GSK-3β (1:1000, cat. no. ab32391, Abcam, UK), anti-phosphorylated GSK-3β (p-GSK-3β, Ser9, 1:1000, cat. no. 5558, Cell Signaling Technology, USA), anti-GAPDH (1:10,000, cat. no. 10494–1-AP, Proteintech), and anti-α-tubulin (1:10,000, cat. no. 11224–1-AP, Proteintech). After washing the membrane with Tris-buffered saline with Tween 20 (TBST), it was incubated with a goat anti-rabbit IgG secondary antibody (1:10,000, cat. no. 20040, Absin, Shanghai, China) at room temperature for 1 h. Following additional washes with TBST, protein bands were visualized using an enhanced chemiluminescence (ECL) reagent kit (Proteintech, Wuhan, China), and images were captured using the SH-Advance523 imaging system (Shenhua Science Technology Co., Ltd., Hangzhou, China). Protein expression levels were quantified by normalizing the intensity of target protein bands to that of GAPDH or α-tubulin.

### Flow cytometry

Cells were cultured until reaching the logarithmic growth phase and subsequently treated with trypsin in the absence of ethylenediaminetetraacetic acid (EDTA). The supernatant was removed by centrifugation, and the cells were washed twice with PBS solution. The cells were then resuspended in 500 μL of pre-cooled 1× buffer to achieve a concentration of 1 × 10^6 cells/mL. 5 μL of Annexin V (Beyotime, Shanghai, China) was added into 100 μL of cell suspension, the mixture was incubated at room temperature in the dark for 15 min, followed by the addition of 2.5 μL of propidium iodide (PI). Flow cytometry (BD, USA) was employed to assess cell apoptosis and cell cycle progression.

### Protein protein interaction (PPI), gene ontology (GO) and Kyoto encyclopedia of genes and genomes (KEGG) pathway analyses

PPI analysis of CCNL1-associated genes was performed using the STRING database (https://string-db.org) to retrieve interaction data. The resulting PPI network was visualized and constructed using Cytoscape software. For functional annotation, GO enrichment analysis (including biological process, cellular component, and molecular function categories) and KEGG pathway enrichment analysis were conducted using the Metascape platform (http://metascape.org). Enriched pathways and functional terms were visualized as bubble plots generated using the R package ggplot2.

### Animal experiments

Six-week-old female Balb/c nude mice were randomly assigned to a negative control (NC) group and a miR-199b-5p mimic group (*n* = 5). 100 μL of H1299 cells suspension, pre-incubated with either miR-NC exosomes or miR-199b-5p mimic exosomes, was administered to the mice via tail vein injection. These exosome injections were conducted at weekly intervals. After 28 days post-injection, *in vivo* imaging was conducted to assess metastasis. D-Luciferin (15 mg/mL, Abcam, UK) was administered intraperitoneally at a dosage of 10 μL/g. Fifteen minutes after post-injection, the mice were anesthetized using 0.7 % pentobarbital sodium, also administered intraperitoneally at a dosage of 10 μL/g. Subsequently, the mice were positioned within a live imaging system (IVIS Spectrum, PerkinElmer, USA) for fluorescence imaging and observation.

### H&E staining

Tumor tissue sections from each mouse group were embedded in paraffin and dewaxed sequentially with xylene. After dewaxing, sections were hydrated through a graded ethanol series (100 %, 95 %, 90 %, 80 %, and 75 %) for rehydration. Hematoxylin staining (Solarbio, Beijing, China) was performed at room temperature for 5 min, followed by eosin staining for 2 min at room temperature. Excess staining solution was removed, and sections were dehydrated again using 90 %, 95 %, and 100 % ethanol for 1 min each. Subsequently, sections were cleared with xylene twice (1 min per immersion). After mounting with neutral balsam, stained sections were observed under a light microscope (Mshot, Guangzhou, China).

### Statistical analysis

Statistical analyses were performed using GraphPad Prism 9.0 software. Data are presented as mean ± standard deviation. All experiments were biologically repeated at least three times. Comparisons between two groups were conducted using Student’s *t*-test. Comparisons among multiple groups were performed using one-way analysis of variance (ANOVA), followed by Fisher's least significant difference (LSD) test. A p-value of <0.05 was considered indicative of a statistically significant difference.

## Results

### Identification of miR-199b-5p in exosomes isolated from serum samples and cells

**Fig. 1:**
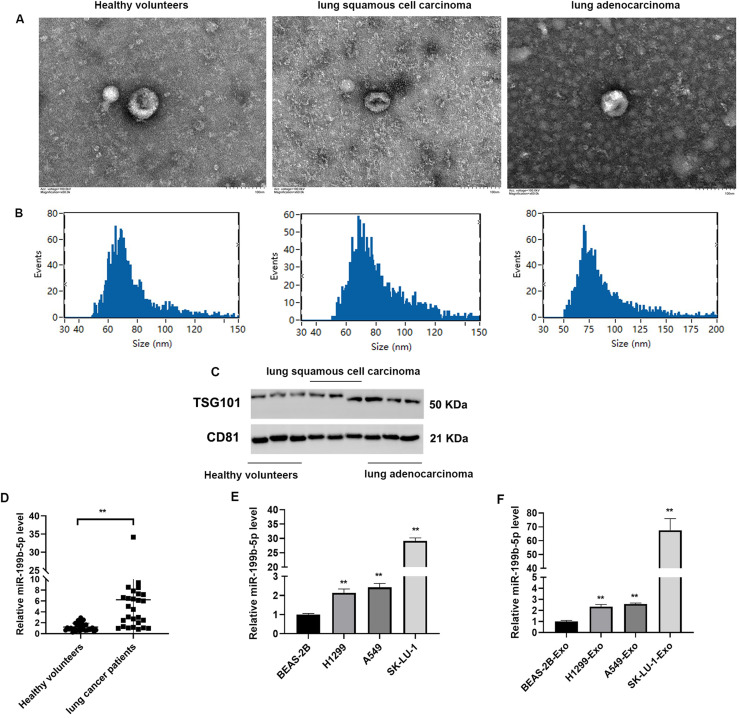
Characterization of exosomes isolated from healthy volunteers and lung cancer patients, and assessment of miR-199b-5p expression across various cell types and exosomes. Exosomal morphology was visualized by transmission electron microscopy (A) and (B); C: Western blot analysis confirmed the expression of canonical exosomal markers TSG101 and CD81; D: QRT-PCR was used to quantify miR-199b-5p expression levels in healthy volunteers and lung cancer patients. Compared to healthy volunteers, **P < 0.01. E: miR-199b-5p expression in different cell lines; F: miR-199b-5p expression in exosomes derived from different cell lines. Compared to BEAS-2B cells or BEAS-2B-, **P < 0.01.

TME was used to characterize the structural features of exosomes. As shown in Fig. 1A and 1B, the isolated exosomes exhibited typical characteristics of exosomes, including a bilayer membrane structure, a particle size ranging from 30 to 100 nm, and a cup-shaped morphology, which are consistent with established exosomal structural attributes. QRT-PCR analysis revealed that miR-199b-5p expression levels showed high consistency among lung cancer patients (Fig. 1C, *P* < 0.01). Furthermore, miR-199b-5p expression was evaluated in BEAS-2B, H1299, A549, and SK-LU-1 cell lines, as well as in exosomes derived from these cells. The findings demonstrated that miR-199b-5p was highly expressed in SK-LU-1 cells (Fig. 1D) and their corresponding exosomes (Fig. 1D, *P* < 0.01).

### Exosomes derived from SK-LU-1 cells enhance motility of lung cancer cells

**Fig. 2:**
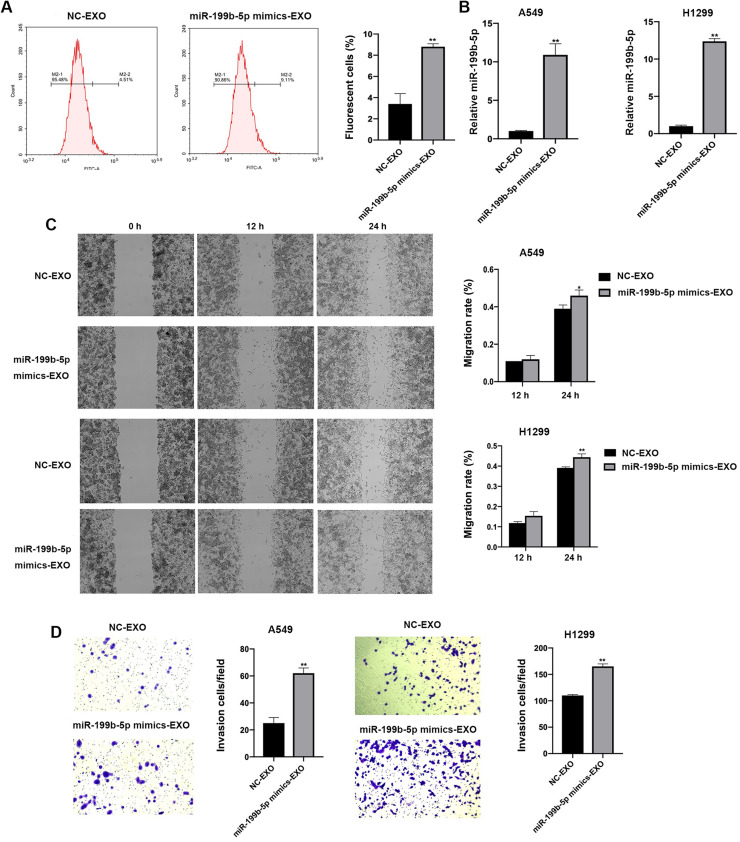
**Exosomes overexpressing miR-199b-5p enhance the motility of NSCLC cells.** A: Flow cytometry analysis showed an increase in the number of fluorescent H1299 cells after incubation with exosomes derived from tumor cells transfected with miR-199b-5p mimics; B: QRT-PCR results confirmed elevated miR-199b-5p expression in A549 and H1299 cells following treatment with the above exosomes; C: Wound scratch healing assay demonstrated enhanced migratory capacity in A549 and H1299 cells treated with exosomes from miR-199b-5p mimic-transfected tumor cells; D: Transwell invasion assay revealed increased invasive potential of A549 and H1299 cells exposed to exosomes derived from miR-199b-5p mimic-transfected cells. Compared to NC-EXO group, *P < 0.05, **P < 0.01.

SK-LU-1 cells were transfected with either negative control (NC) mimics or miR-199b-5p mimics, and exosomes were isolated from these transfected cells. A549 and H1299 NSCLC cells were then incubated with these exosomes (designated as NC-EXO and miR-199b-5p mimic-EXO groups, respectively). Compared to the NC-EXO group, the miR-199b-5p mimic-EXO group showed a significant increase in the number of fluorescent H1299 cells (Fig. 2A). Quantitative real-time PCR analysis confirmed that miR-199b-5p expression levels were significantly upregulated in cells treated with miR-199b-5p mimic-EXO (Fig. 2B, *P* < 0.01). Furthermore, functional assays demonstrated that exosomes derived from miR-199b-5p-overexpressing SK-LU-1 cells enhanced the motility of A549 and H1299 cells, as evidenced by results from wound scratch healing assays (Fig. 2C) and Transwell invasion assays (Fig. 2D, *P* < 0.05).

### Exosomes derived from SK-LU-1 cells promoted the expression of proteins associated with epithelial-mesenchymal transition (EMT) in lung cancer cells

**Fig. 3:**
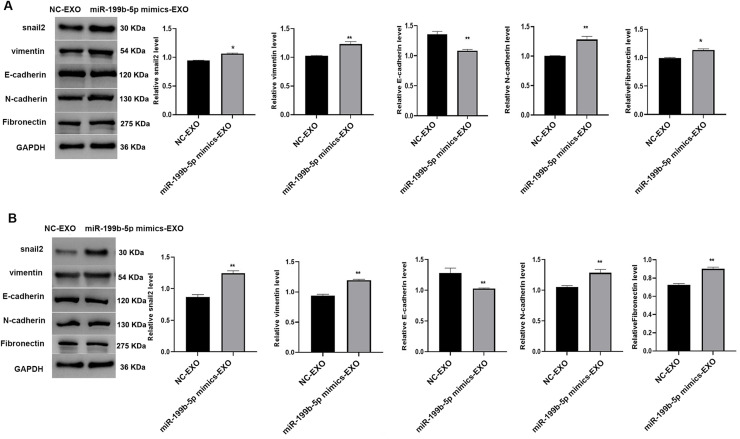
**Exosomes overexpression miR-199b-5p promote epithelial-mesenchymal transition (EMT) in lung cancer cells.** Western blot results revealed altered expression of EMT-related proteins in A549 (A) and H1299 (B) cells exposed to exosomes from miR-199b-5p mimic-transfected tumor cells. Protein expression levels were quantified by densitometry and normalized to GAPDH. Compared to NC-EXO group, *P < 0.05, **P < 0.01.

We examined proteins associated with EMT. The findings indicated that in the miR-199b-5p mimics-EXO group, the expression levels of Snail2, Vimentin, N-cadherin, and Fibronectin were elevated, whereas the level of E-cadherin was reduced (Fig. 3A,B, *P* < 0.05). These results indicated the effect of miR-199b-5p on EMT.

### CCNL1 is a direct target of miR-199b-5p

**Fig. 4:**
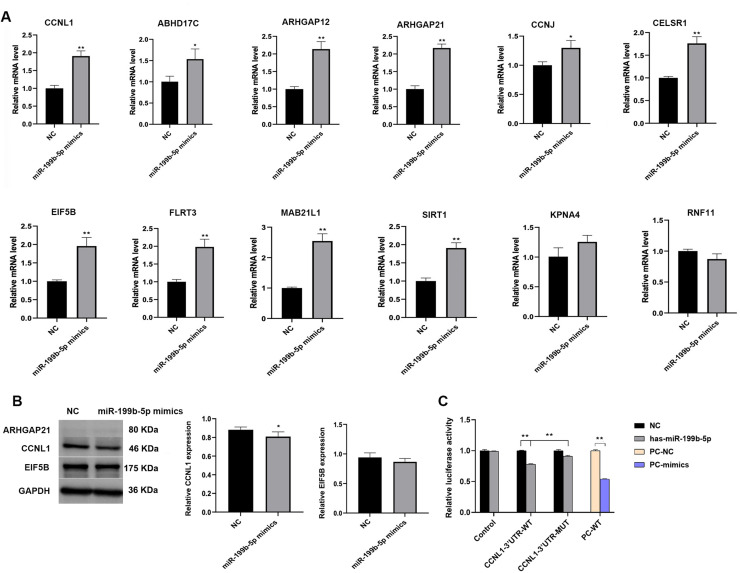
**Identification of miR-199b-5p target genes.** A: QRT-PCR analysis of mRNA expression levels of candidate target genes, including cyclin L1 (CCNL1), ABHD17C, ARHGAP12, ARHGAP21, CCNJ, CELSR1, EIF5B, FLRT3, MAB21L1, SIRT1, KPNA4, and RNF11 in H1299 cells; B: Western blot analysis showing reduced protein expression levels of CCNL1, ARHGAP21, and EIF5B in H1299 cells; Protein expression was quantified by densitometric analysis and normalized to GAPDH. C: Dual luciferase reporter assay confirmed that CCNL1 is a direct target gene of miR-199b-5p Compared to NC group, *P < 0.05, **P < 0.01.

To predict the target genes of miR-199b-5p, we utilized seven miRNA target prediction databases (Diana-microT, Elmmo, Microcosm, miRanda, miRDB, PITA, and TargetScan) and identified a total of 1894 candidate target genes through integrated analysis of their prediction algorithms. Genes that appeared in more than six of these seven databases were selected for further validation, resulting in the identification of 12 candidate genes: CCNL1, ABHD17C (abhydrolase domain-containing 17C), ARHGAP12 (Rho GTPase-activating protein 12), ARHGAP21 (Rho GTPase-activating protein 21), CCNJ (cyclin J), CELSR1 (cadherin EGF LAG seven-pass G-type receptor 1), EIF5B (eukaryotic translation initiation factor 5B), FLRT3 (fibronectin leucine-rich transmembrane protein 3), MAB21L1 (Mab-21 like 1), SIRT1 (silent information regulator sirtuin 1), KPNA4 (karyopherin subunit alpha 4), and RNF11 (ring finger protein 11). Notably, CCNL1 was the only gene predicted by all seven databases. QRT-PCR analysis was performed to assess the expression of these candidate genes. Compared to the NC group, H1299 cells treated with miR-199b-5p mimic-loaded exosomes showed significantly reduced mRNA expression levels of CCNL1, ABHD17C, ARHGAP12, ARHGAP21, CCNJ, CELSR1, EIF5B, FLRT3, MAB21L1, SIRT1, and KPNA4 (*P* < 0.05). Conversely, RNF11 mRNA expression was upregulated in the miR-199b-5p mimic group (Fig. 4A). Western blot analysis revealed undetectable ARHGAP21 protein expression, while CCNL1 and EIF5B protein levels were significantly decreased in the miR-199b-5p mimic group compared to the NC group (Fig. 4B). Furthermore, dual-luciferase reporter assay results confirmed that miR-199b-5p specifically targets CCNL1 (Fig. 4C).

### The overexpression of miR-199b-5p promoted tumor metastasis *in vivo*

**Fig. 5:**
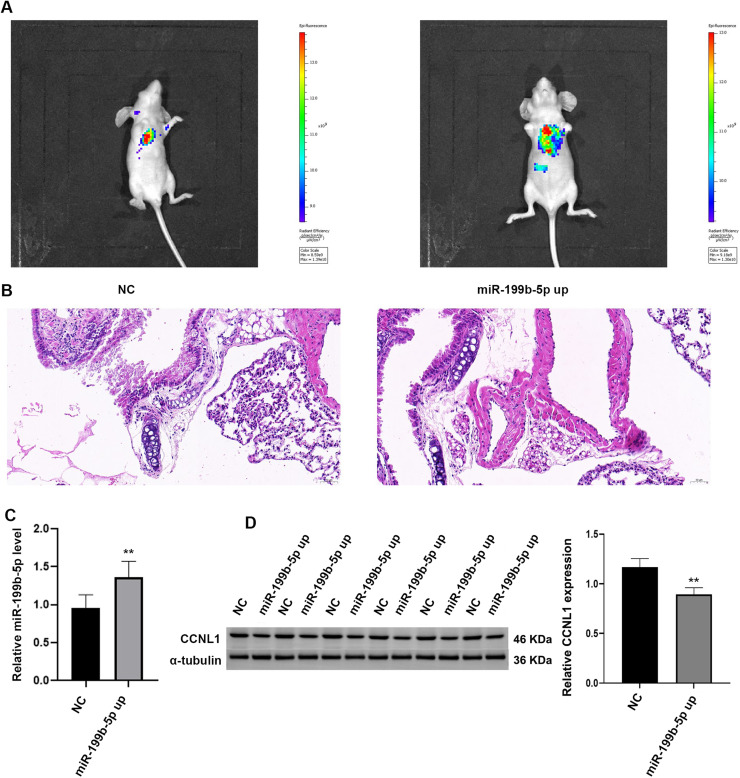
**Overexpression of miR-199b-5p enhances tumor metastasis *in vivo*.** A: *In vivo* tumor metastasis was assessed using a live imaging system, revealing a broader fluorescence signal distribution in the miR-199b-5p up overexpression group compared to the NC group; B: H&E staining confirmed the presence of tumor metastasis in lung tissue; C: Quantification of miR-199b-5p expression levels in lung tissues; D: Evaluation of CCNL1 expression in lung tissues demonstrated that miR-199b-5p overexpression led to downregulated CCNL1 expression. Protein expression was quantified by densitometric analysis and normalized to α-tublin. Compared to NC group, **P < 0.01.

To examine the *in vivo* effects of miR-199b-5p, a murine tumor model was conducted. The fluorescence intensity observed in the miR-199b-5p mimic group was greater than that in the NC group (Fig. 5A). H&E staining revealed that tumor cells metastasized to the lung tissue were densely packed in the NC group. Notably, in the miR-199b-5p mimic group, there was an increase in the number of tumor cells metastasized to the lung tissue (Fig. 5B). Following miR-199b-5p overexpression, the expression level of miR-199b-5p was significantly elevated compared to the NC group, while the expression level of CCNL1 protein was markedly reduced (Fig. 5C, D, *P* < 0.01).

### MiR-199b-5p overexpression promoted H1299 cells proliferation and suppressed cells apoptosis via CCNL1

**Fig. 6:**
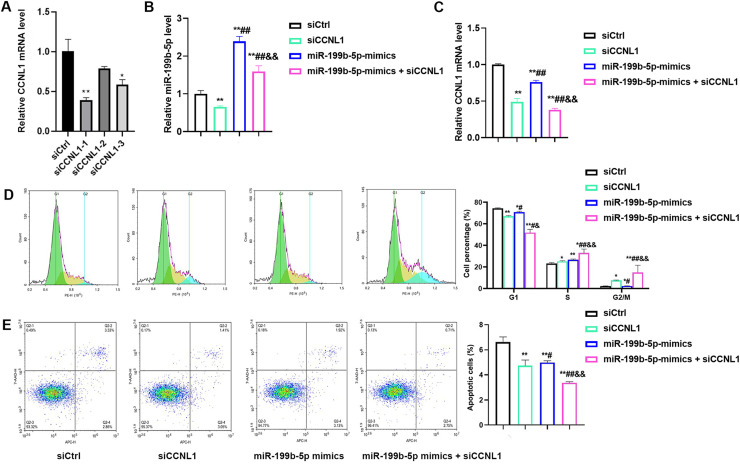
**MiR-199b-5p overexpression promotes proliferation and suppresses apoptosis of H1299 cells via regulation of CCNL1.** A: Three siRNAs targeting CCNL1 were designed, with siCCNL-1 showing the highest knockdown efficiency; B: QRT-PCR analysis of miR-199b-5p expression levels across different groups; C: QRT-PCR quantification of CCNL1 mRNA expression in different groups; D: Flow cytometry analysis was performed to assess cell cycle distribution across different groups; E: Flow cytometry analysis revealed that CCNL1 knockdown reduced the apoptotic rate in H1299 cells. Compared to siCtrl group, *P < 0.05, **P < 0.01; compared to siCCNL1 group, #*P* < 0.05, ##*P* < 0.01, compared to miR-199b-5p mimics group, &*P* < 0.05, &&*P* < 0.01.

To investigate the interactive effects of miR-199b-5p and CCNL1, we designed siRNA to suppress CCNL1 expression. Our findings indicated that siCCNL1–1 was the most effective (Fig. 6A, *P* < 0.01). Subsequently, we categorized the cells into four groups: siCtrl, siCCNL1, miR-199b-5p mimics, and miR-199b-5p mimics + siCCNL1. The results demonstrated that siCCNL1 could suppress miR-199b-5p expression. Notably, miR-199b-5p mimics also suppressed CCNL1 expression (*P* < 0.01). The results of cell cycle revealed that siCCNL1 induced cell arrest in the G1 phase and increased the number of cells in the S phase (Fig. 6B, C, *P* < 0.01). The proportion of cells in the G1 phase further decreased in the miR-199b-5p mimics + siCCNL1 group (Fig. 6D, *P* < 0.05). Additionally, treatment with siCCNL1 and miR-199b-5p mimics reduced cell apoptosis, with the lowest apoptosis rate was observed in the miR-199b-5p mimics + siCCNL1 group (Fig. 6E, *P* < 0.05).

### The overexpression miR-199b-5p partially reversed the inhibitory effect of CCNL1 overexpression

**Fig. 7:**
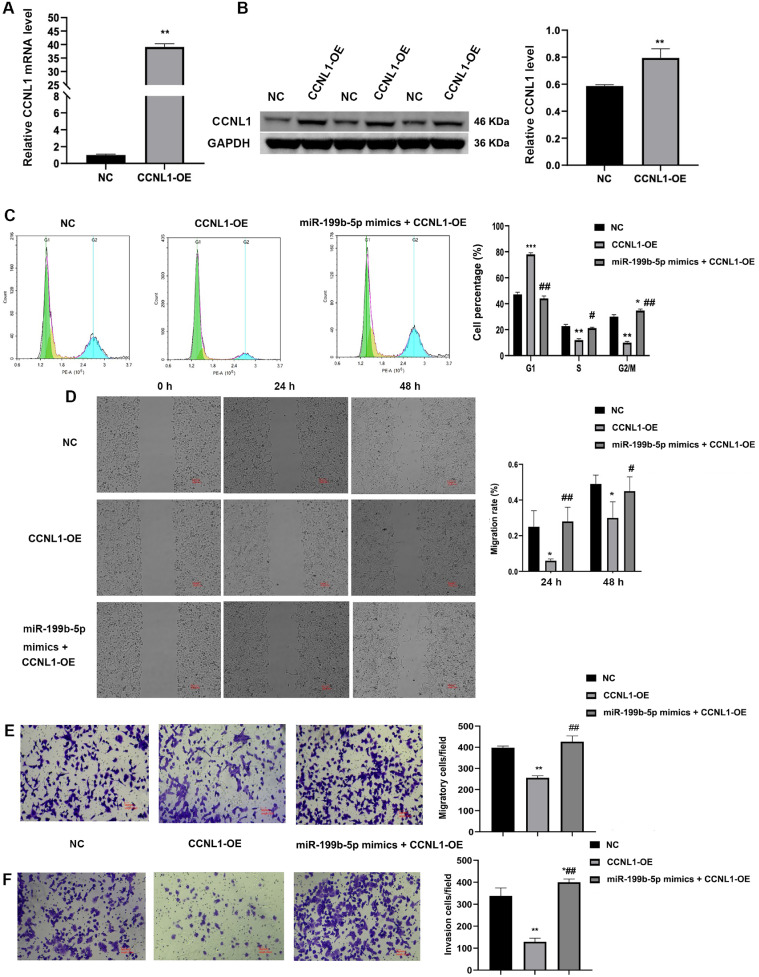
**Overexpression of miR-199b-5p partially attenuates the inhibitory effects of CCNL1 overexpression.** QRT-PCR (A) and western blot (B) analysis confirmed elevated CCNL1 expression following CCNL1 overexpression; C: Flow cytometry analysis was performed to assess cell cycle distribution in H1299 cells treated with exosomes transfected with miR-199b-5p mimics; D: Wound scratch healing assay was used to evaluate the migratory capacity of H1299 cells; Transwell assays were conducted to assess the migratory (E) and invasive (F) capabilities of H1299 cells. Compared to NC group, *P < 0.05, **P < 0.01; compared to CCNL1-OE group, #*P* < 0.05, ##*P* < 0.01.

We conducted an investigation into the effects of CCNL1 overexpression. The findings revealed a significant upregulation in CCNL1 expression level in comparison to the NC group (Fig. 7A,B, *P* < 0.01). Cell cycle results demonstrated that CCNL1 overexpression resulted in an increased proportion of cells in the G1 phase, while concurrently reducing cells in the S phase (Fig. 7C, *P* < 0.01). Moreover, the overexpression of CCNL1 markedly inhibited cellular migration and invasion capabilities (Fig. 7D-F, *P* < 0.01). Notably, the administration of miR-199b-5p mimics partially mitigated the inhibitory effects associated with CCNL1 overexpression.

### Downstream mechanism of miR-199b-5p targeting CCNL1

**Fig. 8:**
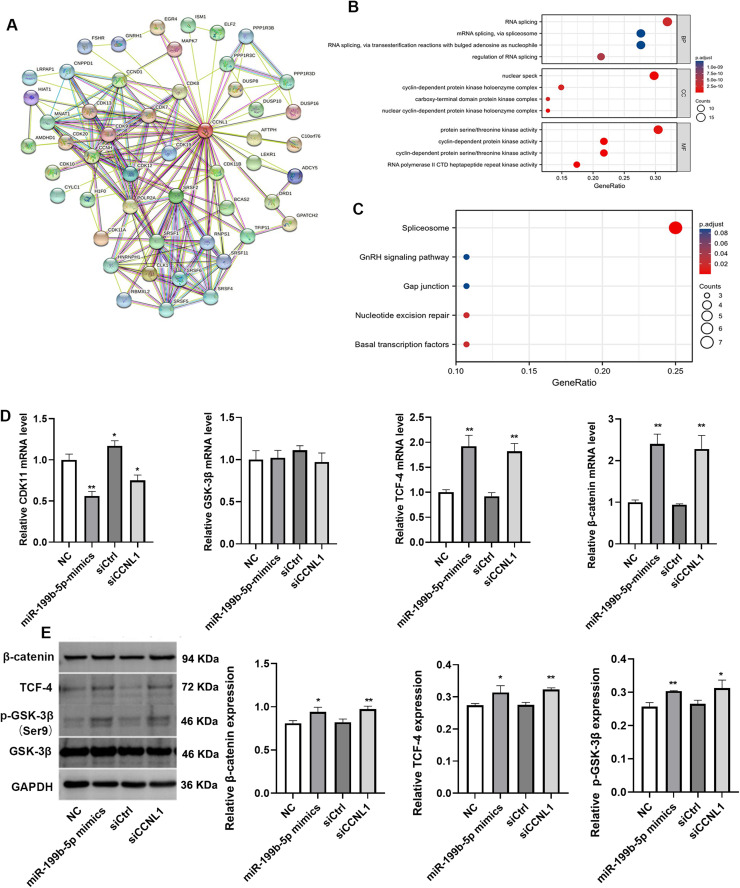
**Overexpression of miR-199b-5p enhances activation of the Wnt/β-catenin signaling pathway by downregulating CCNL1 expression.** A: Protein protein interaction (PPI) analysis of genes associated with CCNL1; Gene Ontology (GO) functional enrichment (B) and Kyoto Encyclopedia of Genes and Genomes (KEGG) pathway enrichment analysis (C) of 50 CCNL1-related genes; D: Quantitative real-time PCR (qRT-PCR) analysis of mRNA expression levels of CDK11, TCF-4, β-catenin, and GSK-3β; E: Western blot analysis to evaluate the protein expression levels of TCF-4, β-catenin, p-GSK-3β, and total GSK-3β. Compared to NC or siCtrl group, *P < 0.05, **P < 0.01.

To explore the downstream mechanisms of CCNL1, we performed GO functional enrichment and KEGG pathway enrichment analyses on 50 CCNL1-associated genes identified from the PPI network. The top five enriched pathways, ranked by gene ratio, are illustrated in Fig. 8A-C. GO functional analysis revealed that these genes are significantly enriched in biological processes including RNA splicing, nuclear speck formation, and protein serine/threonine kinase activity. KEGG pathway analysis further indicated significant enrichment in the spliceosome pathway. Subsequent experiments evaluated the expression of key molecules involved in these pathways, including CDK11, TCF-4, β-catenin, and GSK-3β. QRT-PCR results showed that transfection with miR-199b-5p mimics or siCCNL1 significantly decreased CDK11 mRNA expression while upregulating TCF-4 and β-catenin mRNA levels (Fig. 8D, *P* < 0.05). Consistent with these findings, Western blot analysis demonstrated that miR-199b-5p mimics or siCCNL1 markedly increased the protein expression of TCF-4, β-catenin, and p-GSK-3β (Fig. 8E, *P* < 0.05).

## Discussion

Exosomes are disc-shaped vesicles with diameters ranging from 40–120 nm, secreted by various cell types, including tumor cells [20]. In this study, we isolated exosomes from healthy volunteers and NSCLC patients, detecting particle sizes between 30 and 100 nm and a characteristic cup-shaped morphology, consistent with known exosome structures. Recent studies have demonstrated that exosomal miRNAs play significant roles in angiogenesis, cell metastasis, immune regulation, and other process related to lung cancer [21,22]. Our findings revealed elevated expression levels of miR-199b-5p in lung cancer patients. Specifically, the expression level of miR-199b-5p was significantly higher in NSCLC cells compared to BEAS-2B cells. These results are consistent with previous study, which demonstrated high expression of miR-199b-5p in tumor tissues and cells [15].

The role of miR-199b-5p in various cancer types remains a subject of debate. Chen et al. demonstrated that miR-199b-5p is significantly overexpressed in gastric cancer tissues and correlates with poor prognosis in gastric cancer patients [12]. Conversely, Ren et al. identified miR-199b-5p as a tumor-suppressive miRNA in papillary thyroid carcinoma cells [23]. These findings suggest that miR-199b-5p may exert context-dependent roles across different cancer types. In our study, we observed that exosomes containing overexpressed miR-199b-5p significantly enhanced the motility of A549 and H1299 cells. Additionally, these exosomes promoted EMT by upregulating the expression of Snail2, vimentin, N-cadherin, and fibronection, while downregulating E-cadherin expression- all of which are well-recognized EMT markers [24]. To elucidate the underlying mechanisms of miR-199b-5p, we utilized seven miRNA target prediction databases to identify 1894 potential target genes. We selected genes that appeared in more than six of the seven databases, with CCNL1 emerging as the only gene predicted across all seven databases. A dual-luciferase reporter assay confirmed that miR-199b-5p specifically targets CCNL1.

Muller et al. reported that CCNL1, a potential oncogene, is localized to the frequently amplified 3q25–28 chromosomal region in human head and neck squamous cell carcinomas [25]. Zeng et al. demonstrated that CCNL1 overexpression promotes prostate cancer cell proliferation and is inversely correlated with miR-5195–3p expression in prostate cancer tissues [26]. Yang et al. proposed that the loss of CCNL1 induces cellular resistance to gemcitabine treatment [27]. However, the role of CCNL1 as a candidate oncogene in head and neck cancer remains incompletely elucidated [28]. Its functional relevance in other cancer types also remains poorly defined. In our study, CCNL1 knockdown recapitulated the effect of miR-199b-5p overexpression, whereas CCNL1 overexpression suppressed cell migration and invasion. To further explore the downstream mechanisms of CCNL1, we performed PPI analysis, GO functional enrichment, and KEGG pathway enrichment analysis. These analyses revealed that CCNL1 is associated with CDK11, TCF-4, β-catenin, and GSK-3β.

Cyclin dependent kinases (CDKs) constitute a major class of serine/threonine protein kinases that are activated upon binding to cyclin regulatory subunits [29]. Recent studies have demonstrated that the CDK11/cyclin L complex functions in cell cycle regulation, transcription, and RNA splicing [30]. The Wnt/β-catenin signaling pathway is implicated in various aspects of cancer progression, including cell proliferation, invasion, and malignant transformation [31]. CDK11 has been identified as a key player in the Wnt/β-catenin signaling pathway through its role in regulating microtubule stability [32]. In our study, we demonstrated that miR-199b-5p overexpressing exosomes inhibit CDK11 mRNA expression while upregulating the expression of TCF-4, and β-catenin. Western blot analysis further corroborated that miR-199b-5p mimics or siCCNL1 stimulate the protein expression of TCF-4, β-catenin, and p-GSK-3β. Zhao et al. reported that miR-199b-5p likely mediates its effects on osteogenesis in human bone marrow stromal cells via the GSK-3β/β-catenin signaling pathway [33]. Our recent research has also confirmed that miR-199b-5p exerts regulatory effects on NSCLC through hypoxia inducible factor 1 subunit alpha inhibitor (HIF1AN) [34]. In the present study, we further elucidated the underlying mechanism by which CCNL1 is associated with Wnt/β-catenin signaling. However, our study has several limitations. Firstly, we only evaluated the effect of exosomes carrying the miR-199b-5p mimic, and the impact of miR-199b-5p knockdown remains unassessed. Secondly, our investigation was restricted to miR-199b-5p overexpression exosomes derived from SK-LU-1 cells, with no exploration of such exosomes from other NSCLC cell lines. Finally, additional clinical studies are required to validate the correlation between miR-199b-5p and CCNL1 expression.

Herein, our study confirms that miR-199b-5p plays a significant role in NSCLC. Exosomes carrying the miR-199b-5p mimic enhance NSCLC cell motility and EMT, with CCNL1 identified as a direct target for miR-199b-5p The underlying mechanism is linked to the Wnt/β-catenin signaling. Our findings identify a novel therapeutic target for NSCLC and provide experimental evidence supporting the potential of exosome-based therapy in NSCLC treatment.

## Ethics approval and consent to participate

All experiments were approved by the Medical Ethics Review of The Second People's Hospital of Wuhu City (Approve number: 2022 [8]).

## Consent for publication

Not applicable

## Availability of data and materials

All data is available from the authors upon request.

## Funding

This work was funded by the Anhui science and technology development fund projects guided by China government in 2021(2020b07030008), Wuhu Science and Technology Projects (2022jc76), The Key Project Funding from Wuhu Health Commission (WHWJ2023z014), The Anhui Provincial Health Commission General Project (AHWJ2023A20165), and “Ai Fei” Public Welfare Project for Clinical Research and Innovation in Tumor Therapy (AF-004).

## CRediT authorship contribution statement

**Bangzhu Liu:** Writing – review & editing, Writing – original draft, Validation, Methodology, Funding acquisition, Conceptualization. **Yan Rui:** Writing – review & editing, Supervision, Software, Methodology, Formal analysis. **Miao Li:** Writing – review & editing, Validation, Investigation, Formal analysis. **Linian Huang:** Writing – review & editing, Writing – original draft, Funding acquisition, Conceptualization.

## Declaration of competing interest

The authors declare that they have no known competing financial interests or personal relationships that could have appeared to influence the work reported in this paper.

## Appendix Supplementary materials

Image, application 1

Image, application 2
