# Supplementary material for: Tumor-derived exosomal miR-199b-5p promotes proliferation and epithelial-mesenchymal transition in non-small cell lung cancer by targeting CCNL1
Source: Transl Oncol. 2025 Oct 16;62:102564. doi: 10.1016/j.tranon.2025.102564 (PMC12554135; doi:10.1016/j.tranon.2025.102564)
Supplement: Supplementary file 1 [file mmc1.docx]

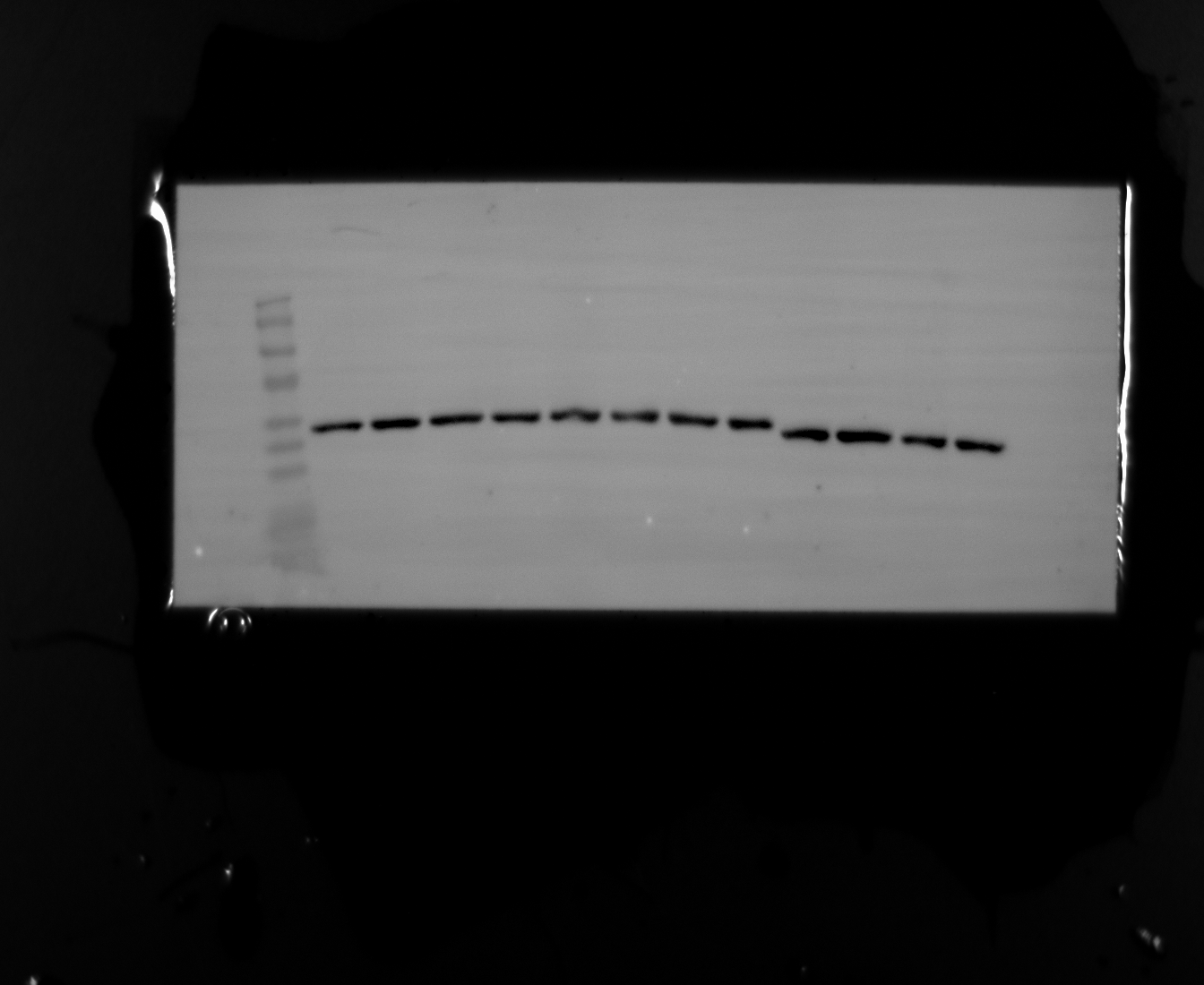


TSG101-Figure1


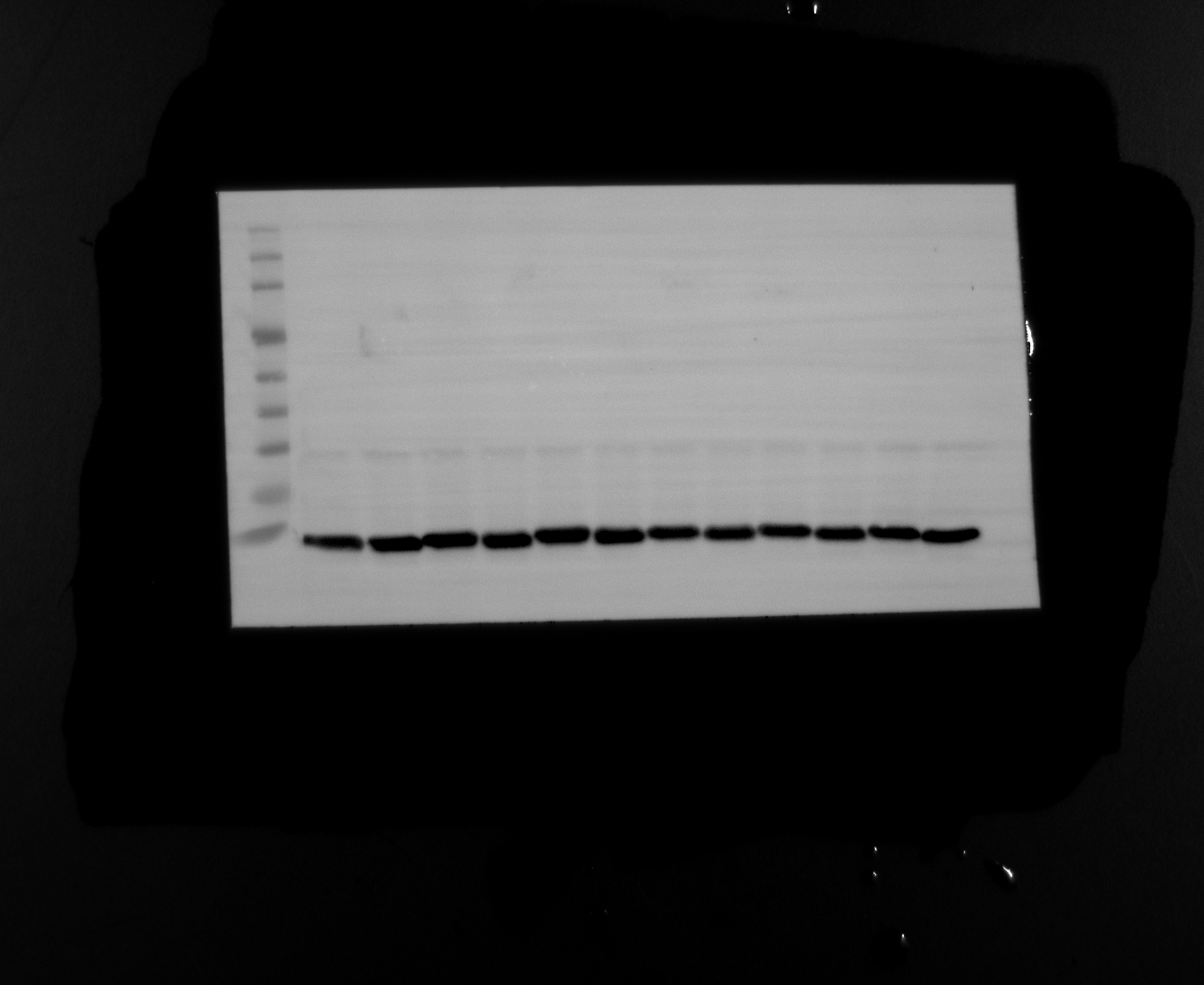


CD81-Figure1


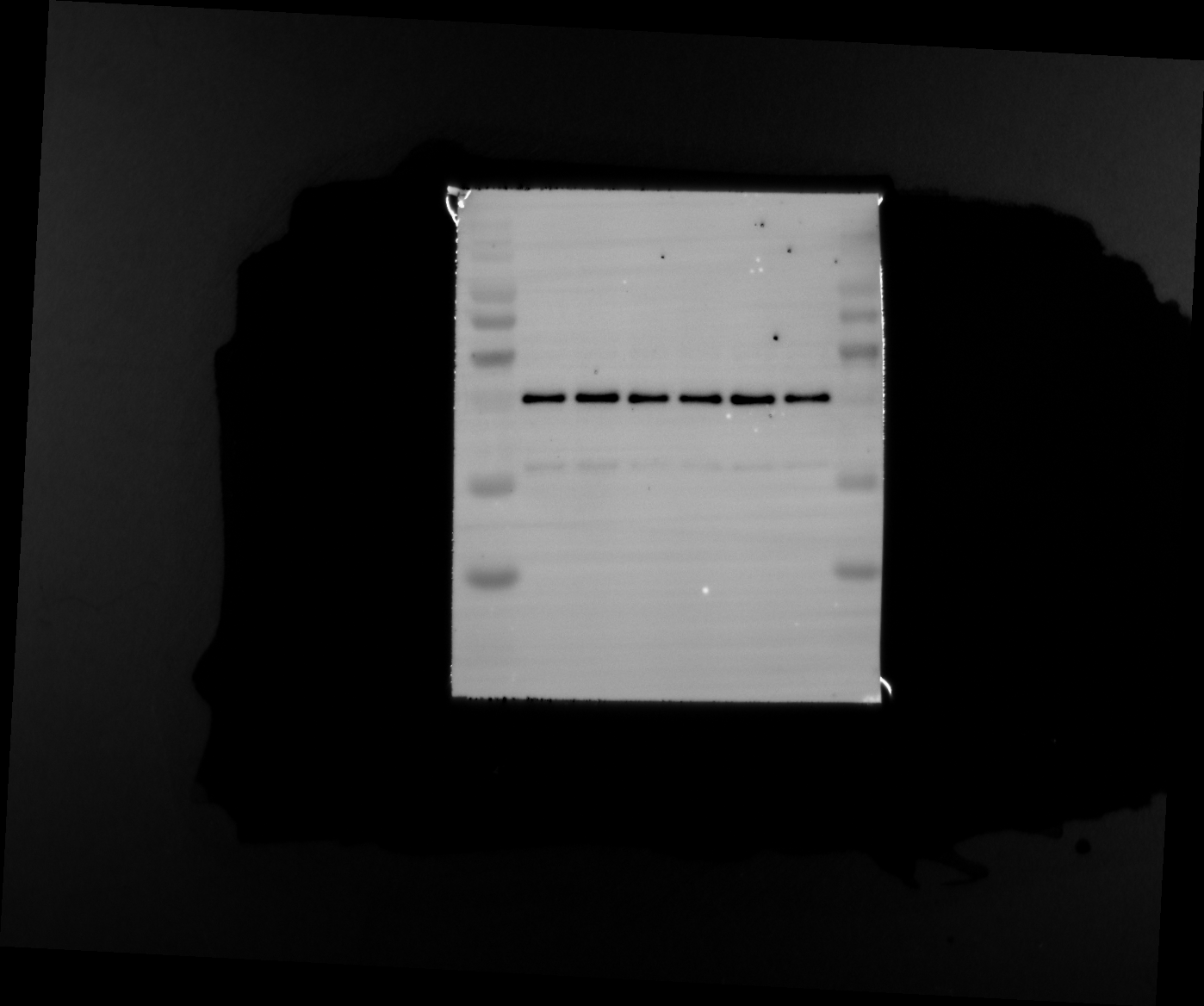


GAPDH-Figure3A


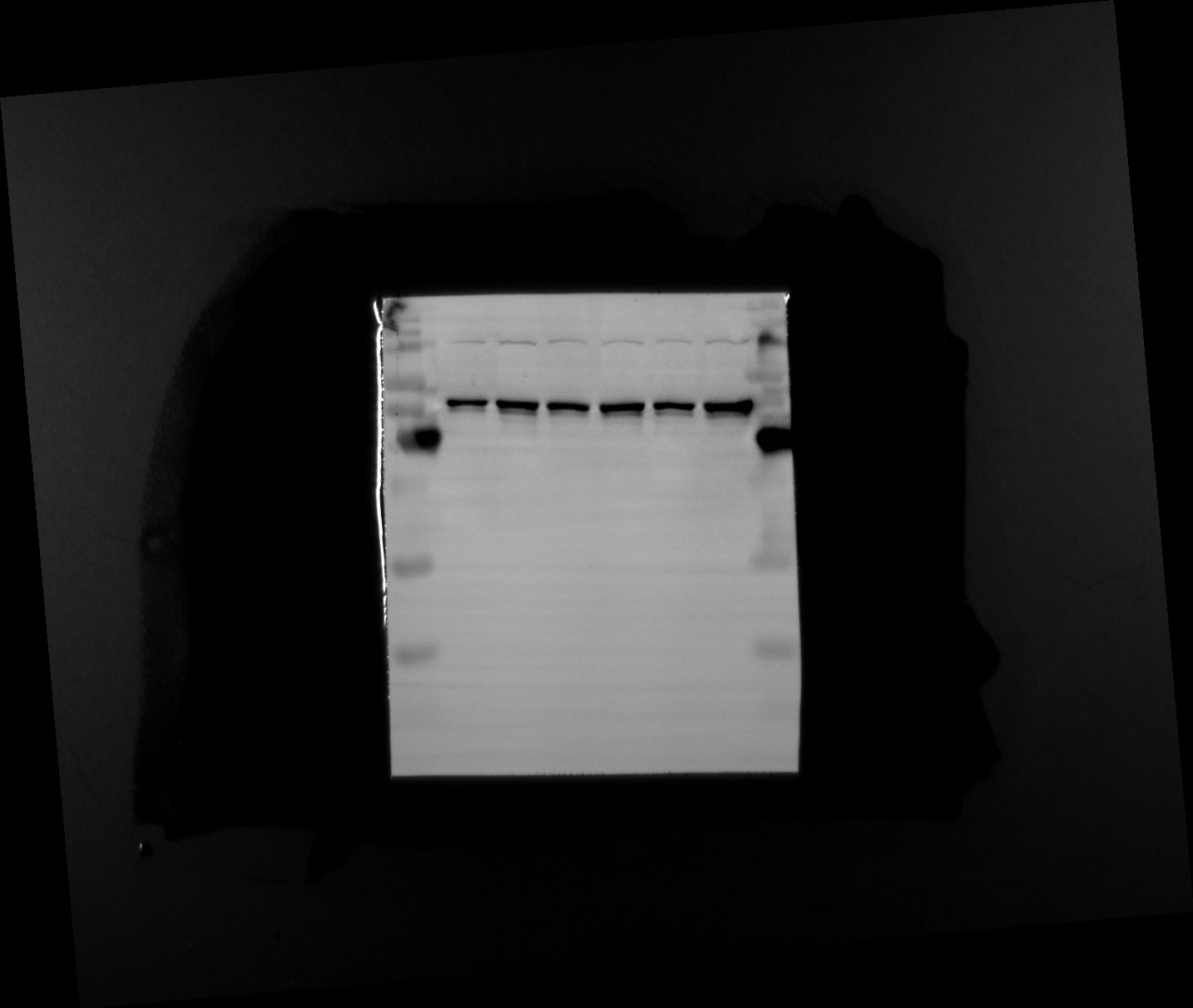


Vimentin-Figure3A


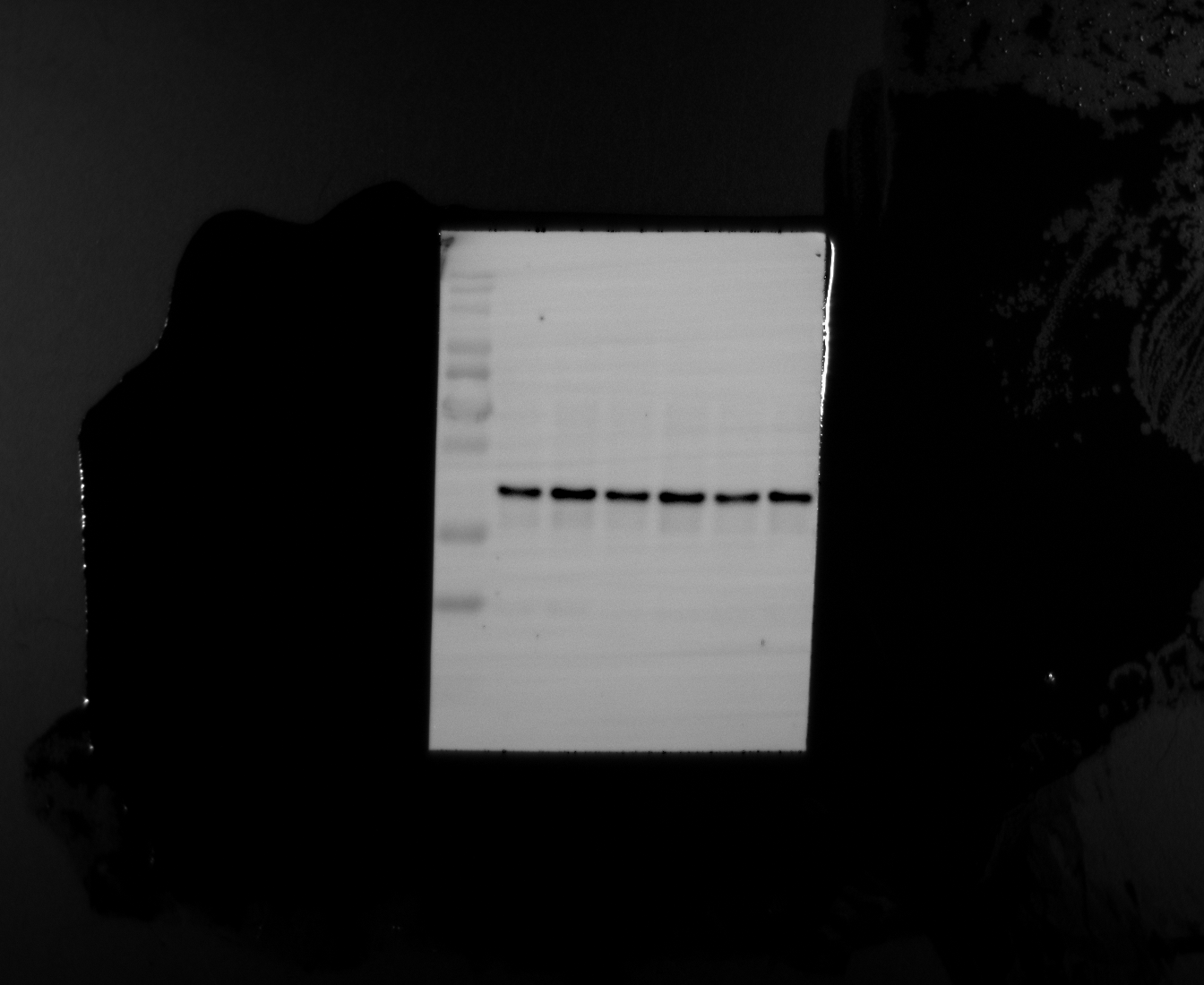


Snail-Figure3A


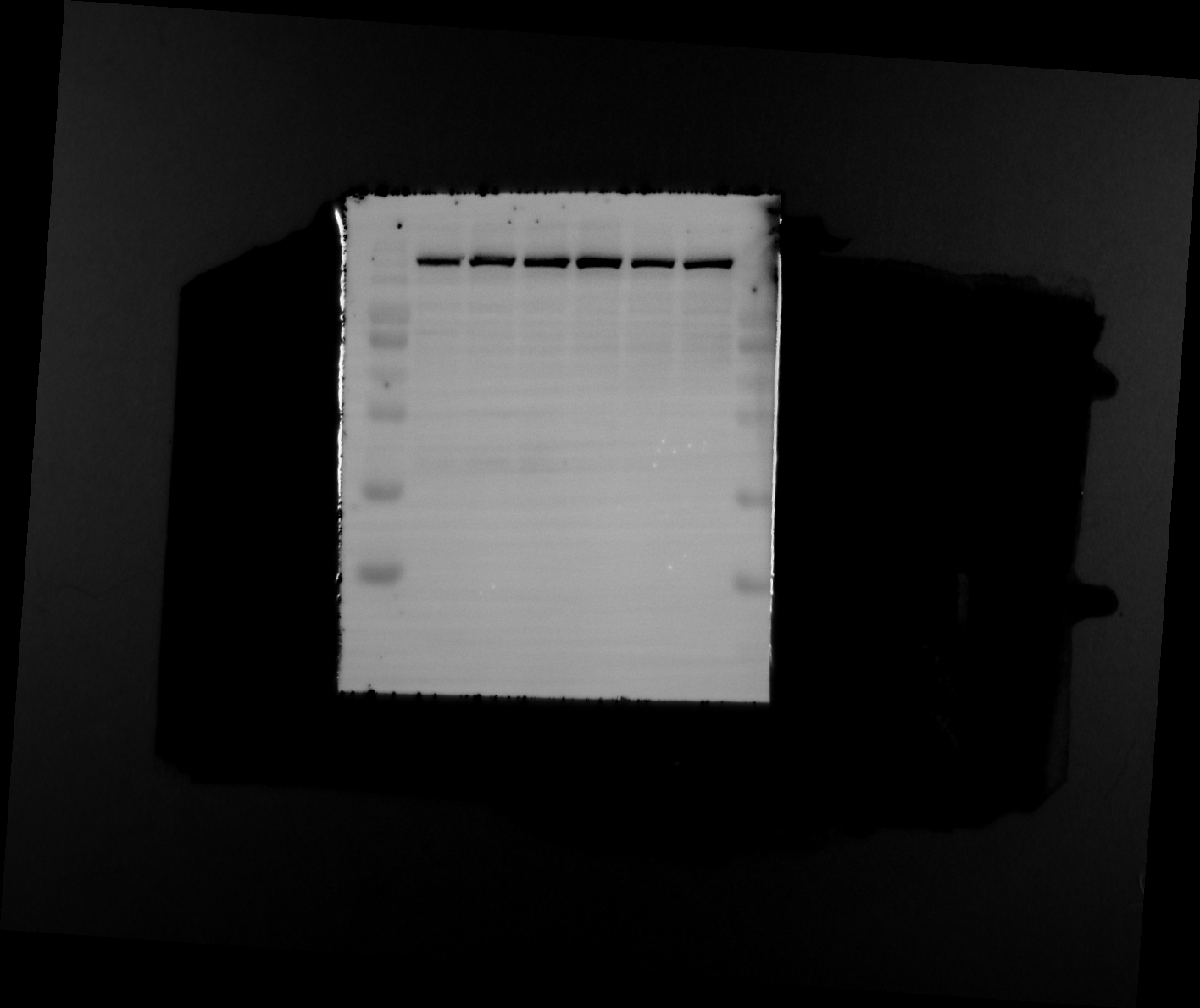


1. Cadherin-Figure3A


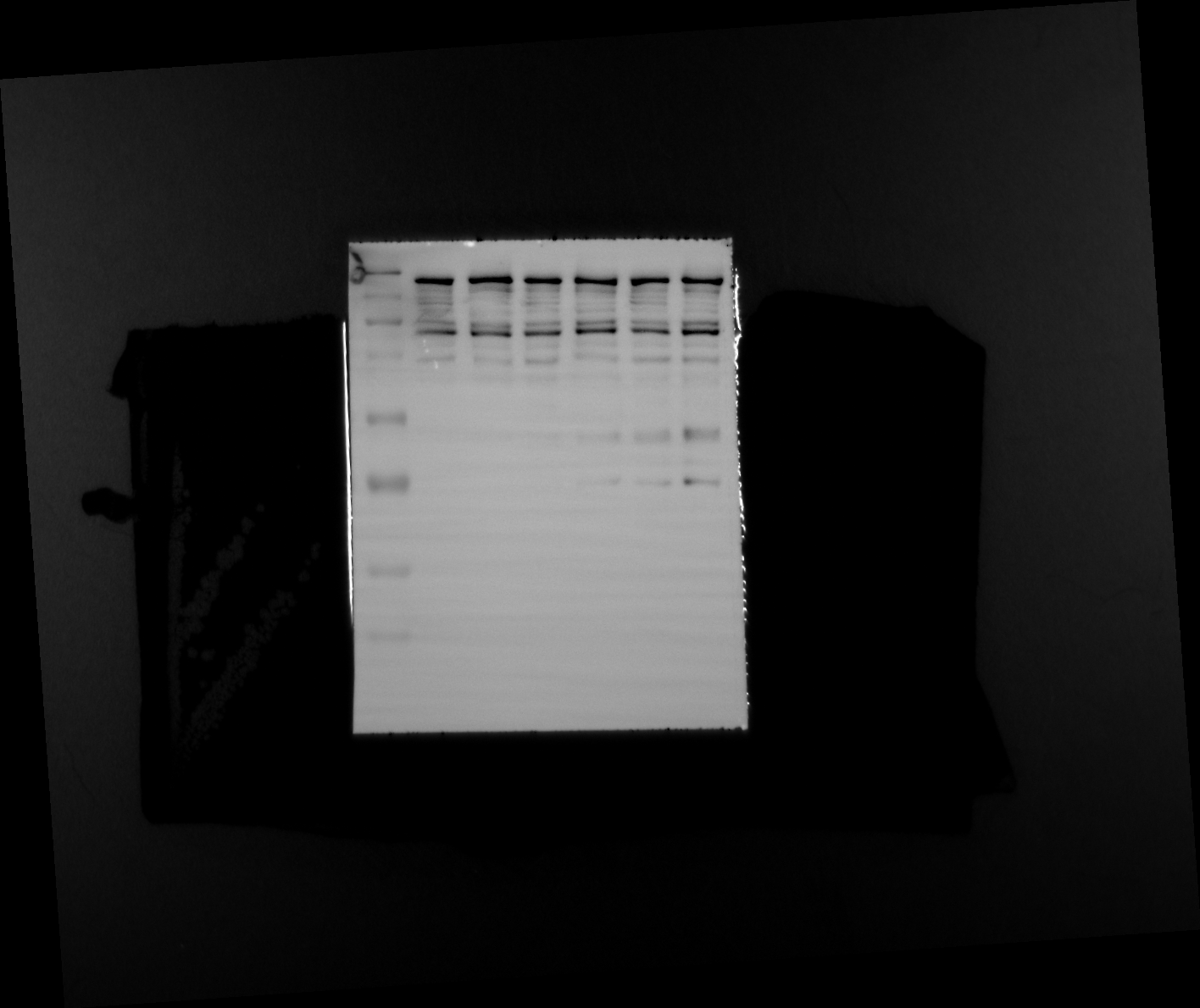


Fibronectin-Figure3A


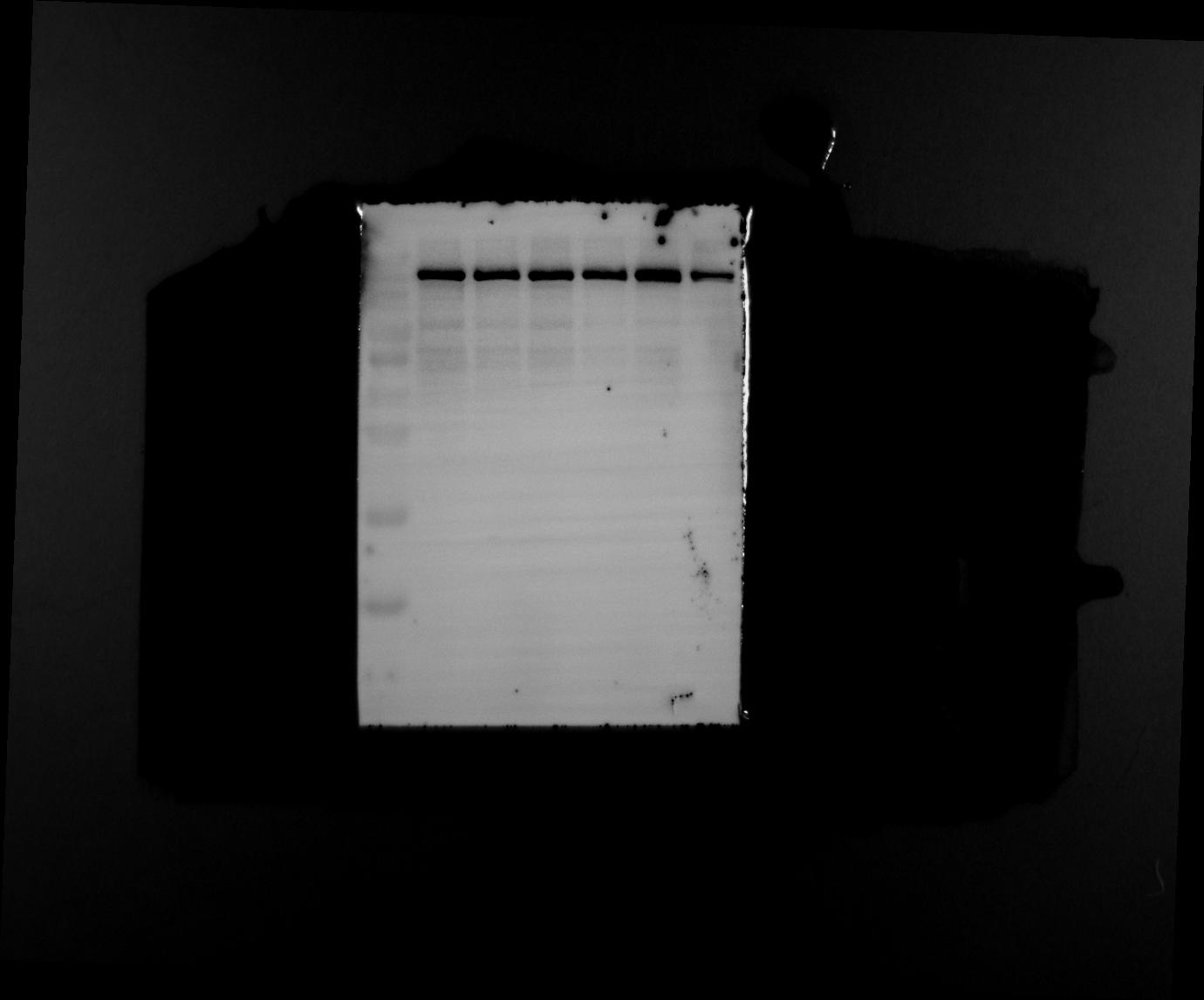


E-Cadherin-Figure3A


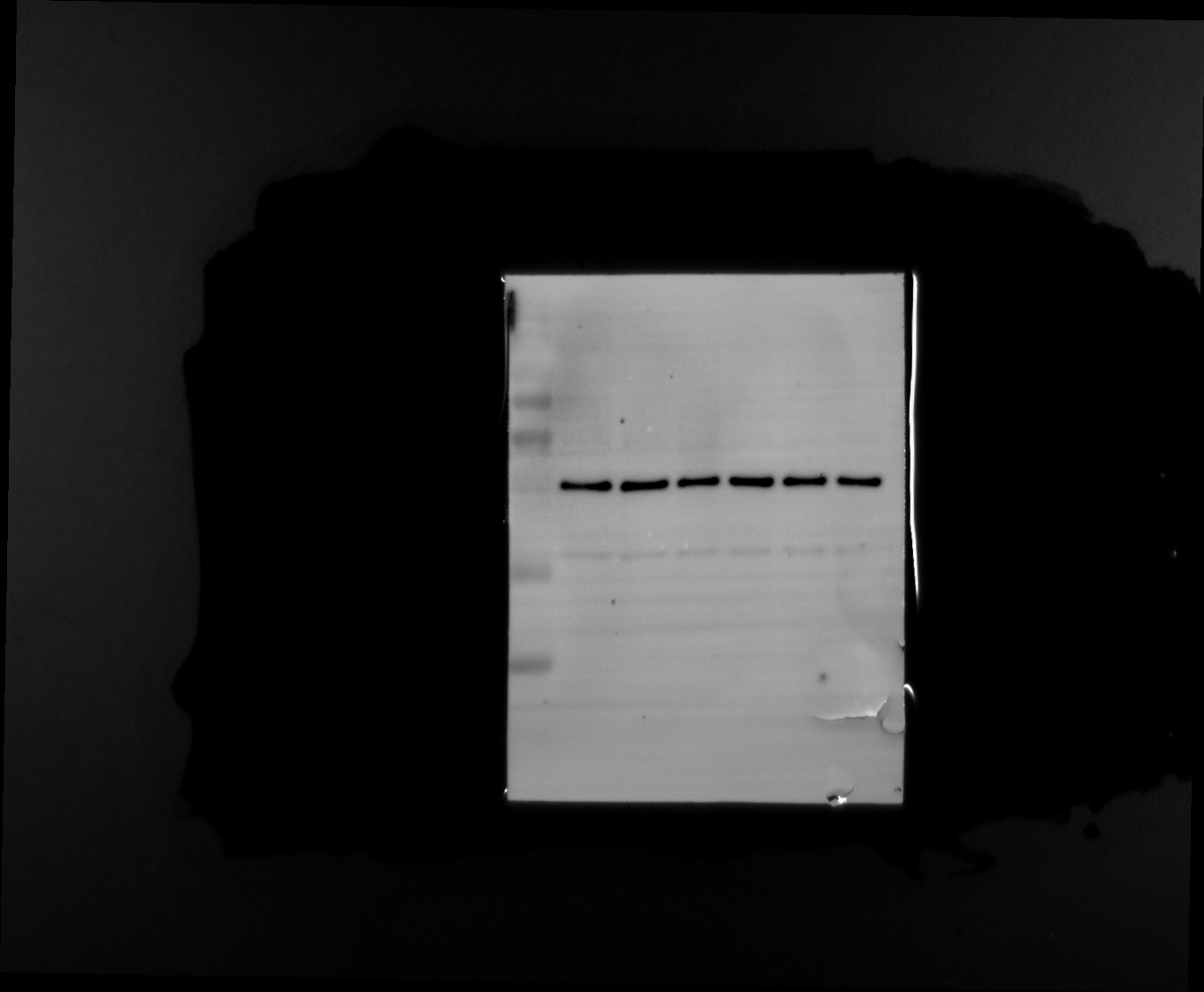


GAPDH-Figure3B


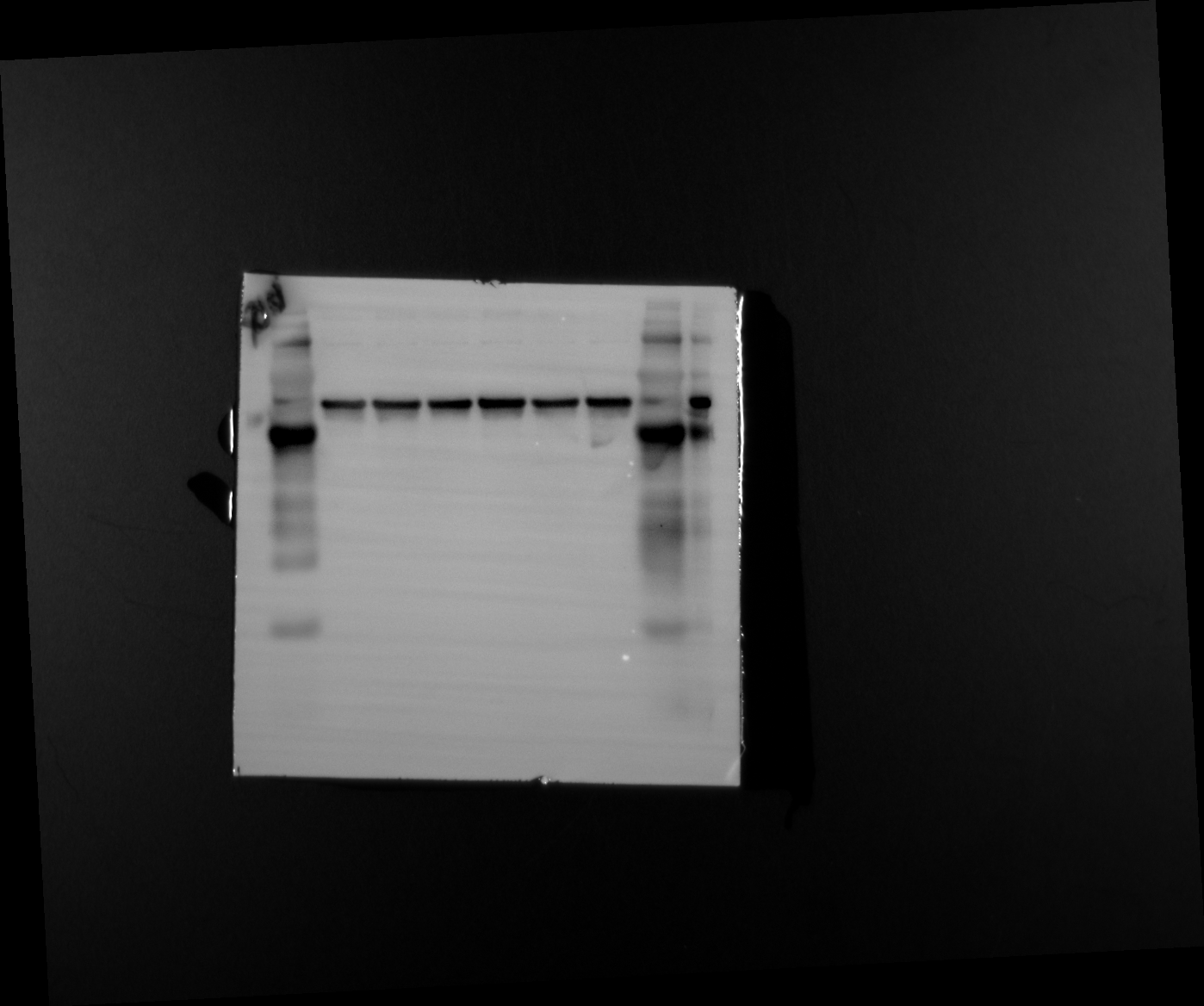


Vimentin-Figure3B


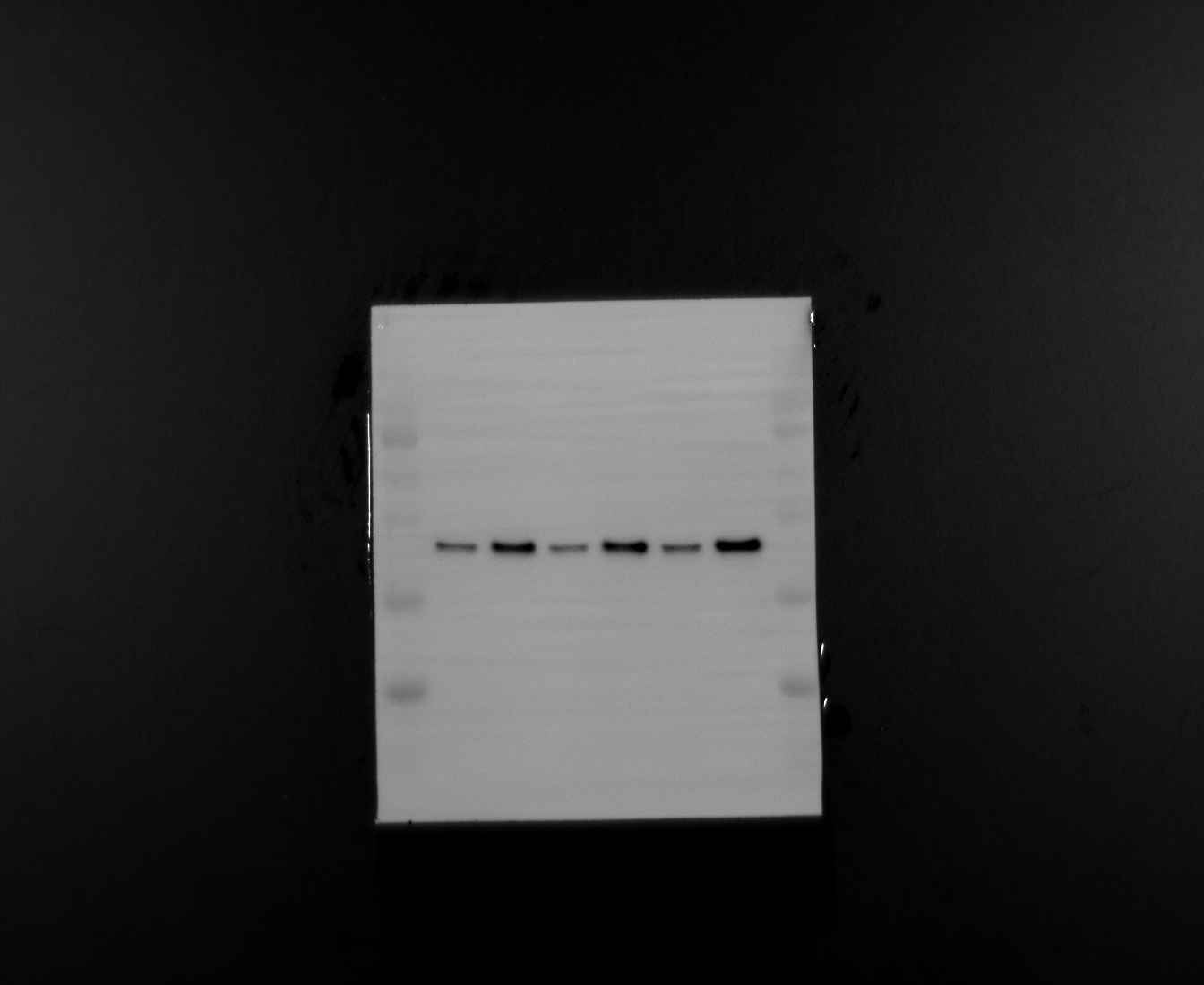


Snail-Figure3B


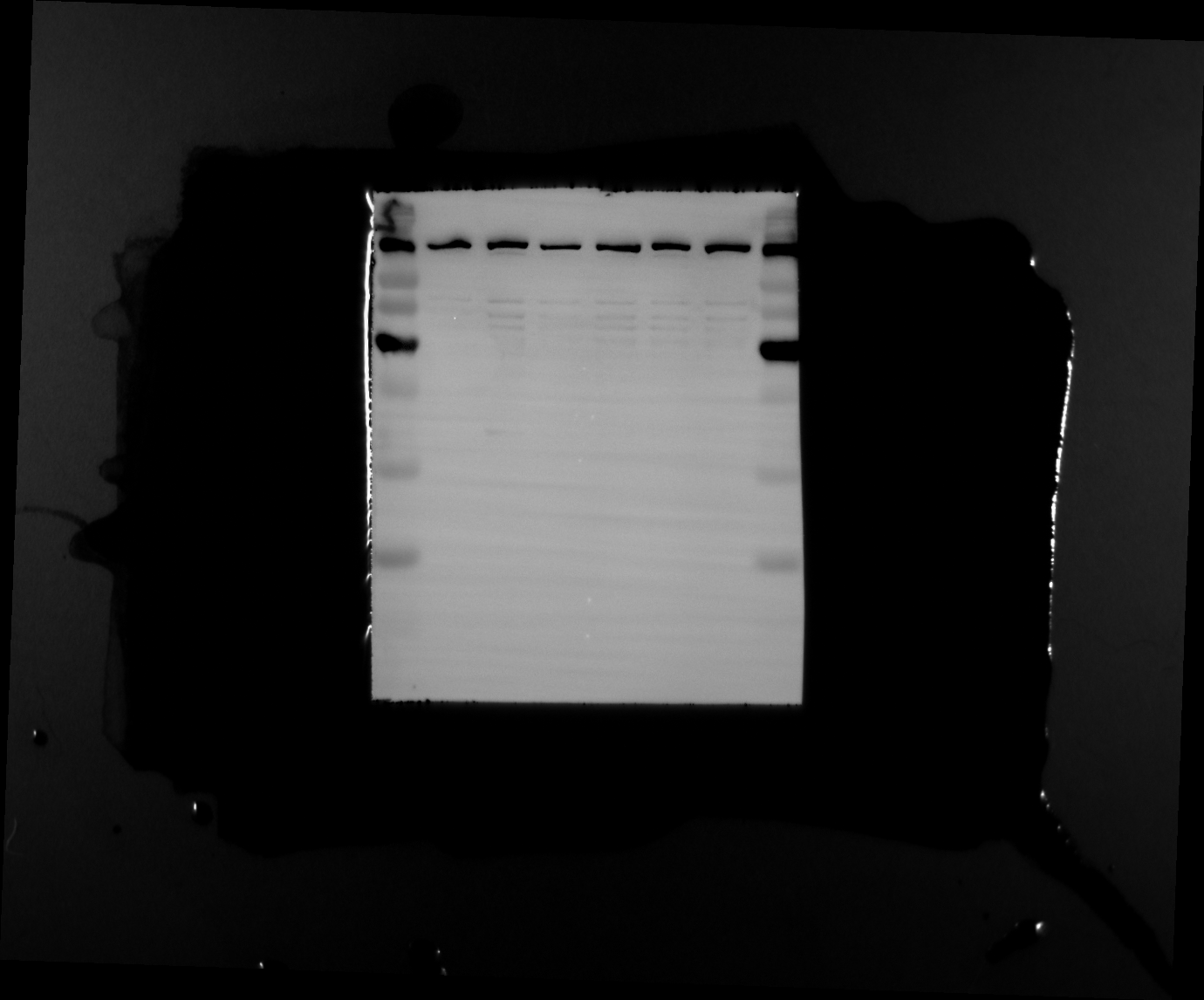


N-Cadherin-Figure3B


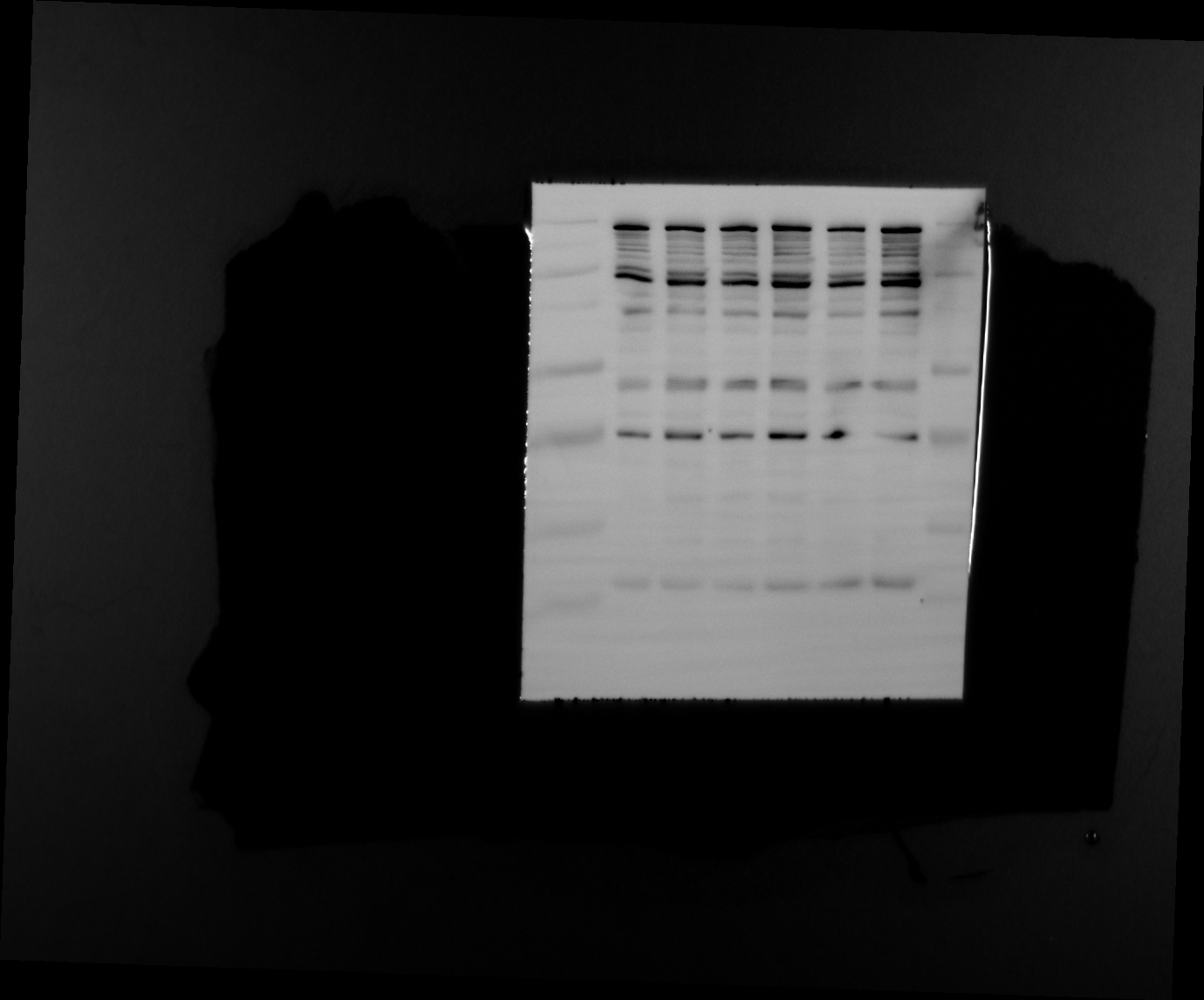


Fibronectin-Figure3B


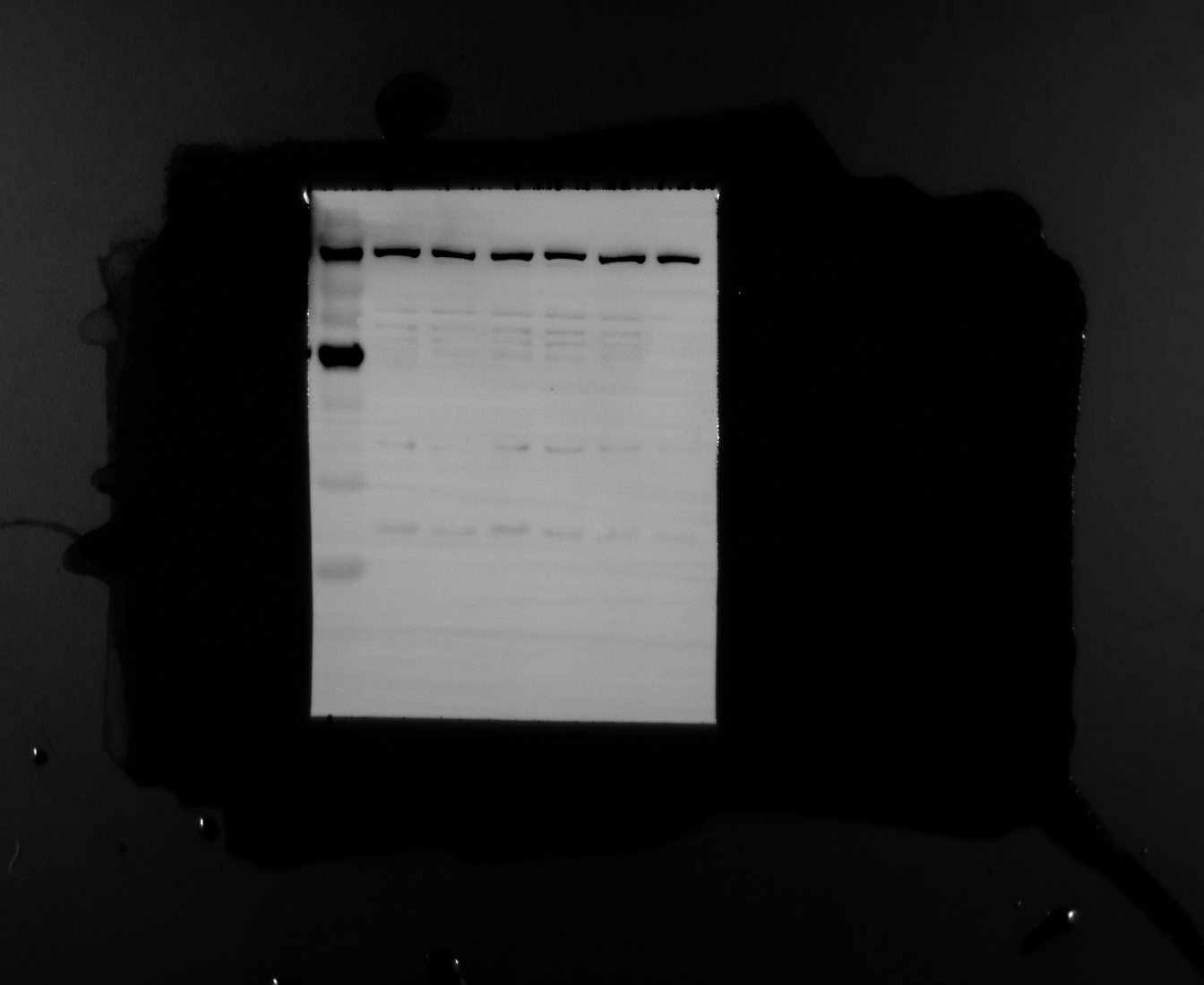


1. Cadherin-Figure3B


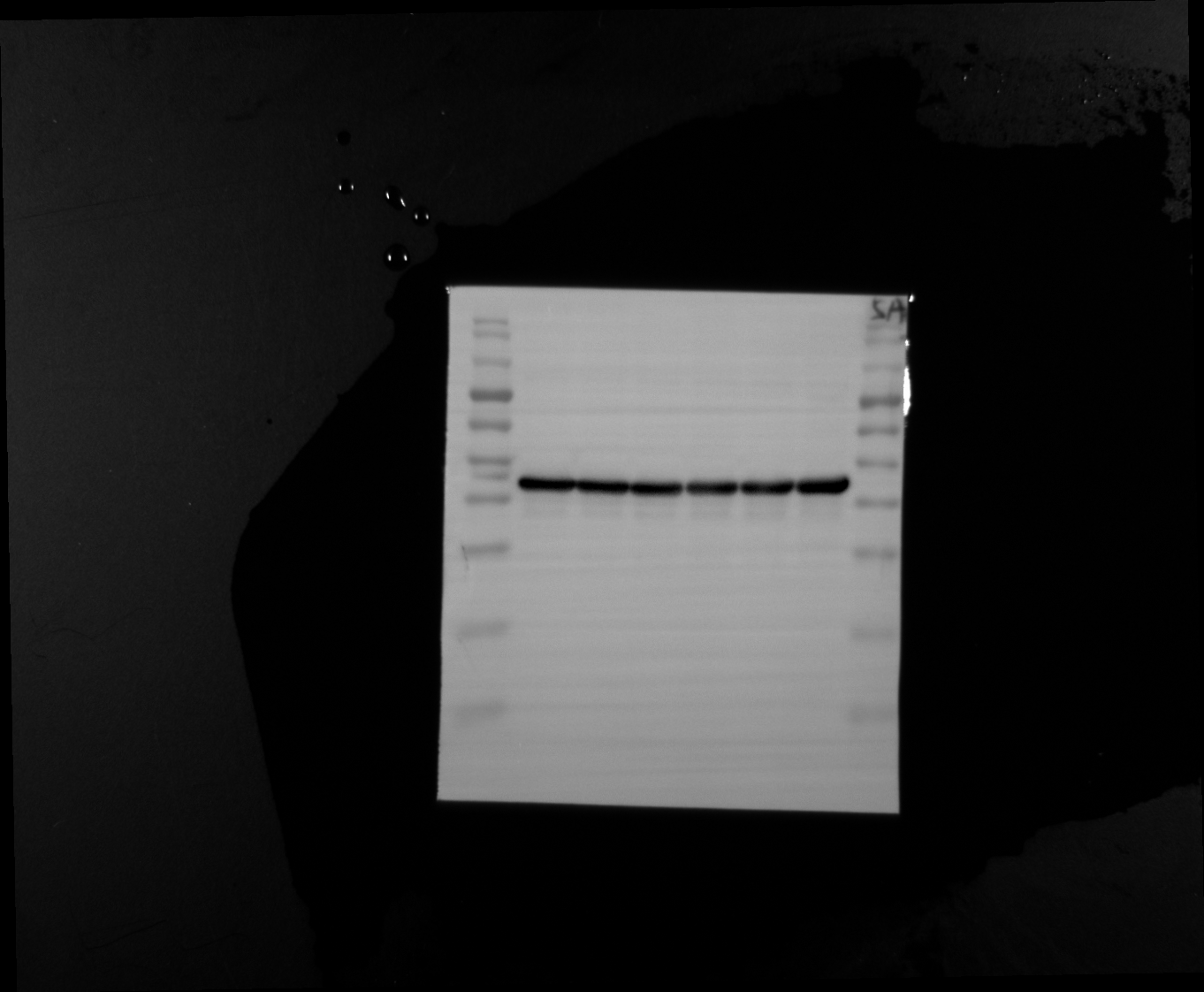


GAPDH-Figure4


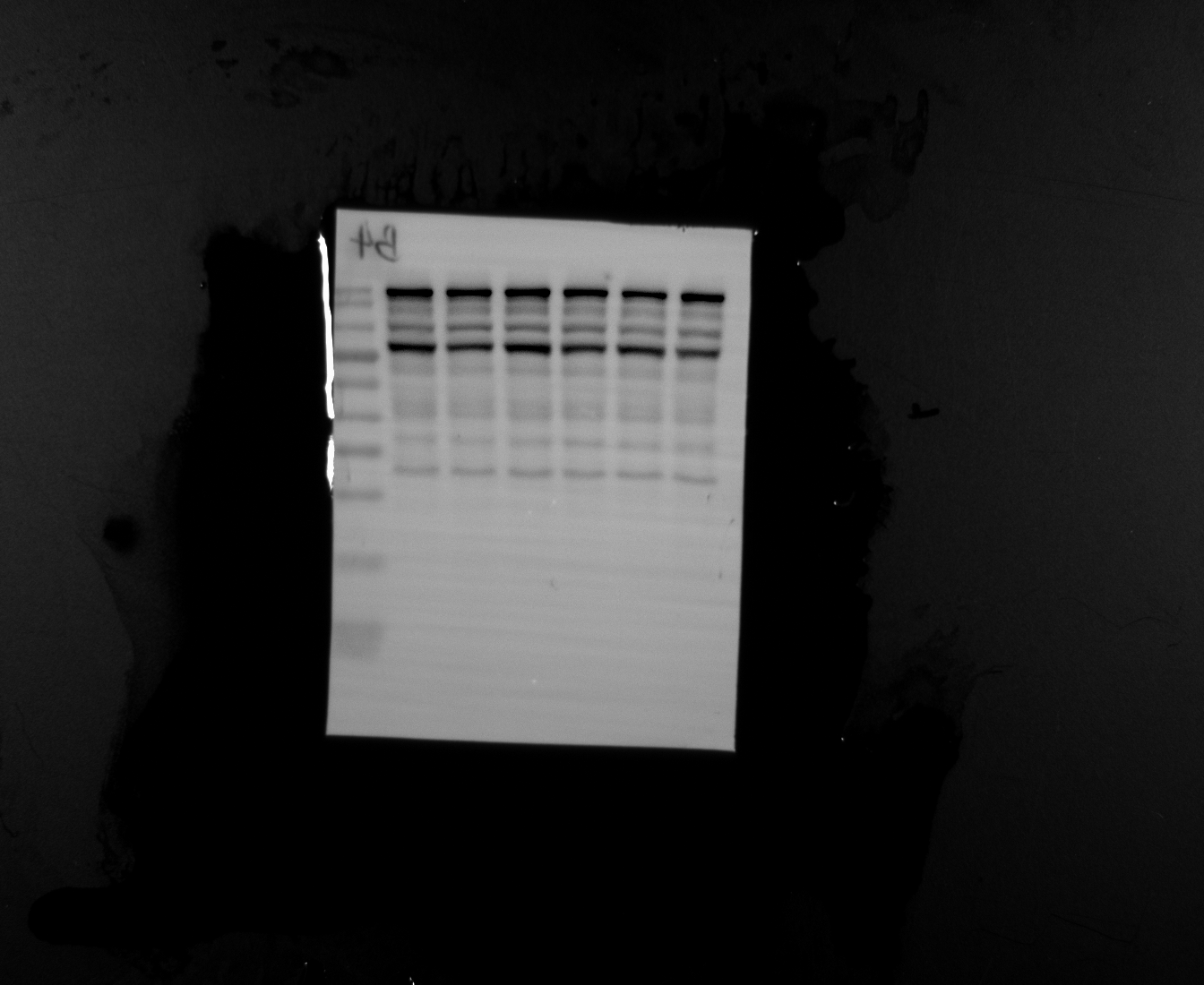


EIF5B-Figure4


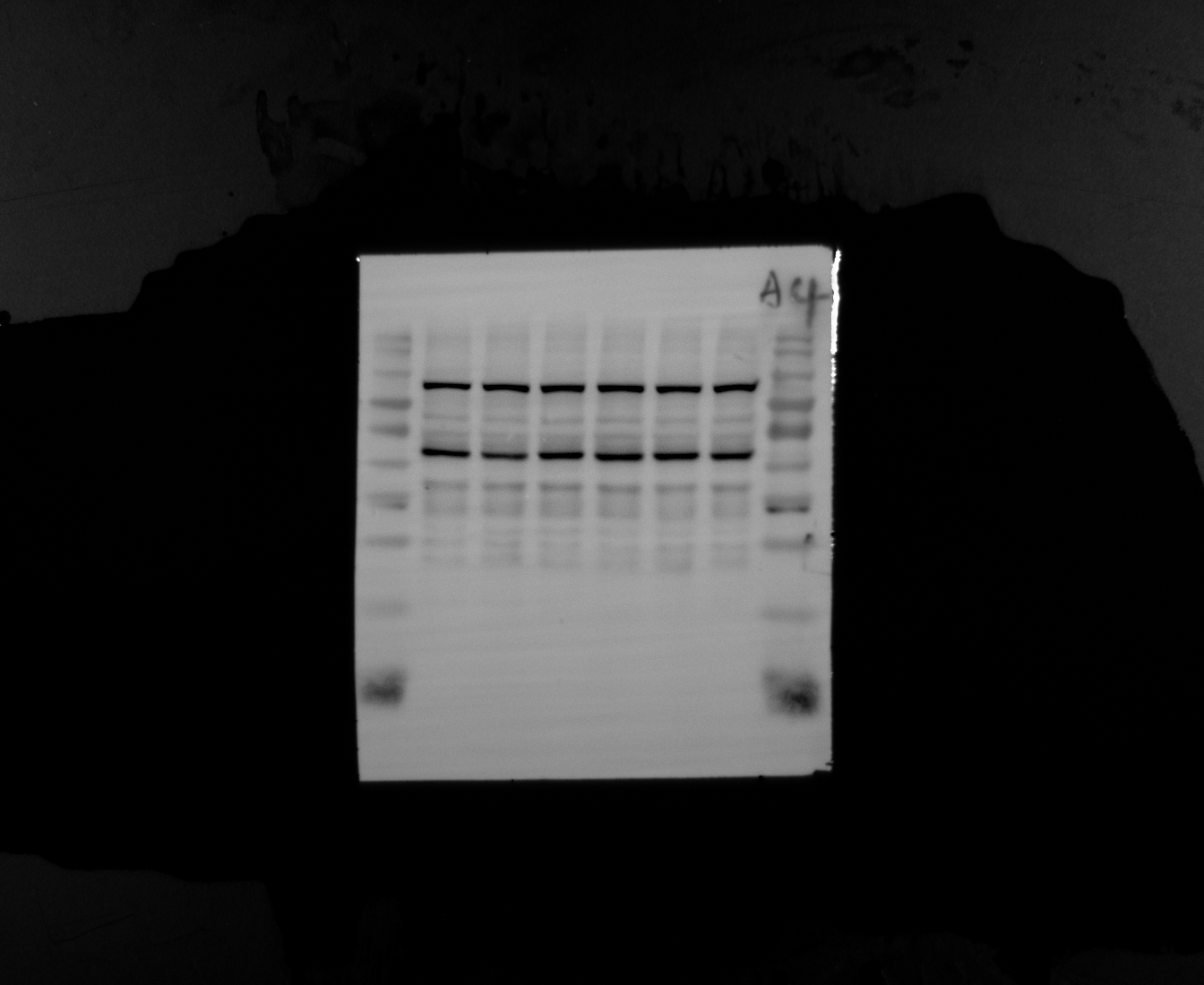


CCNL1-Figure4


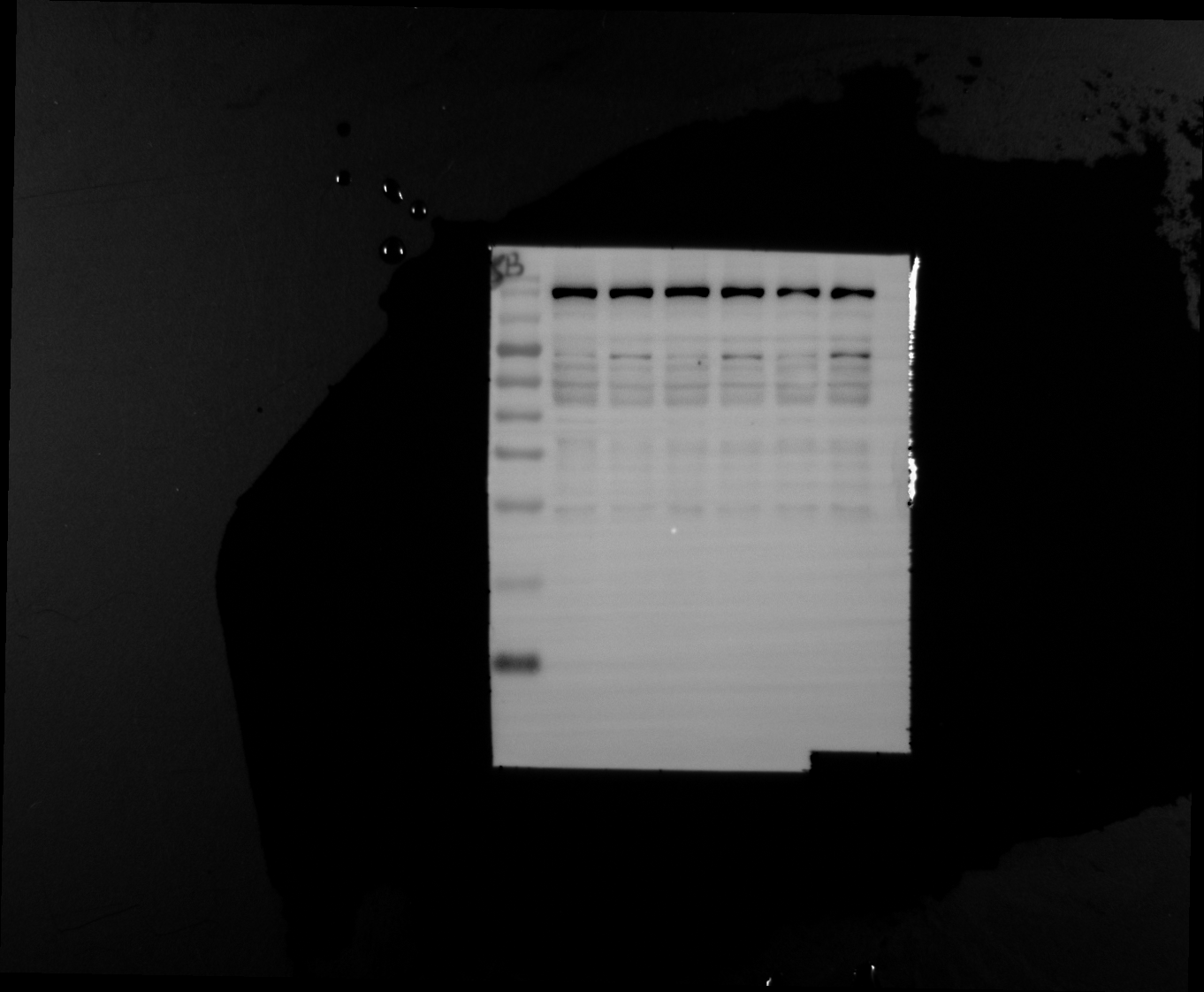


ARHGAP21-Figure4


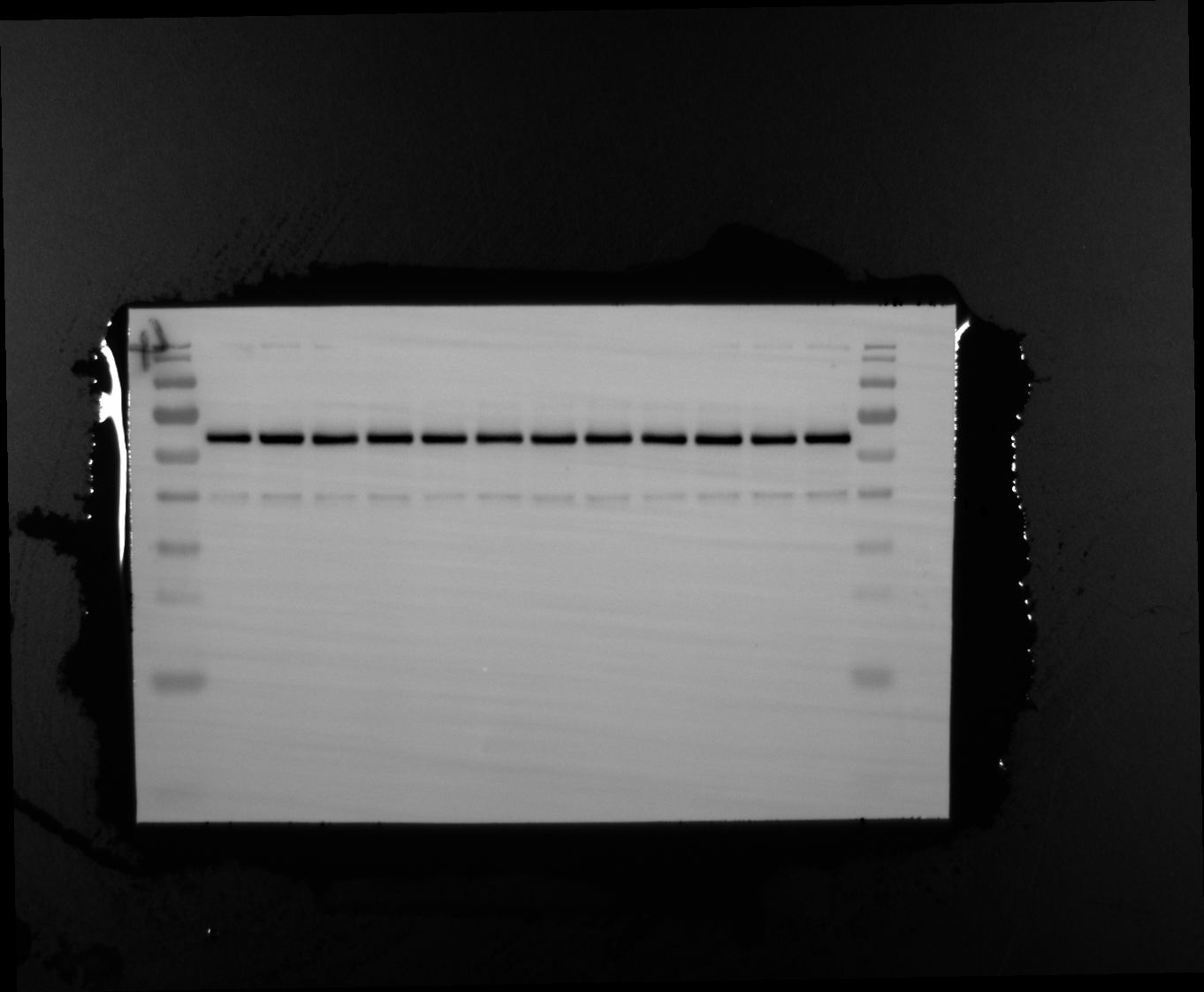


CCNL1-Figure5


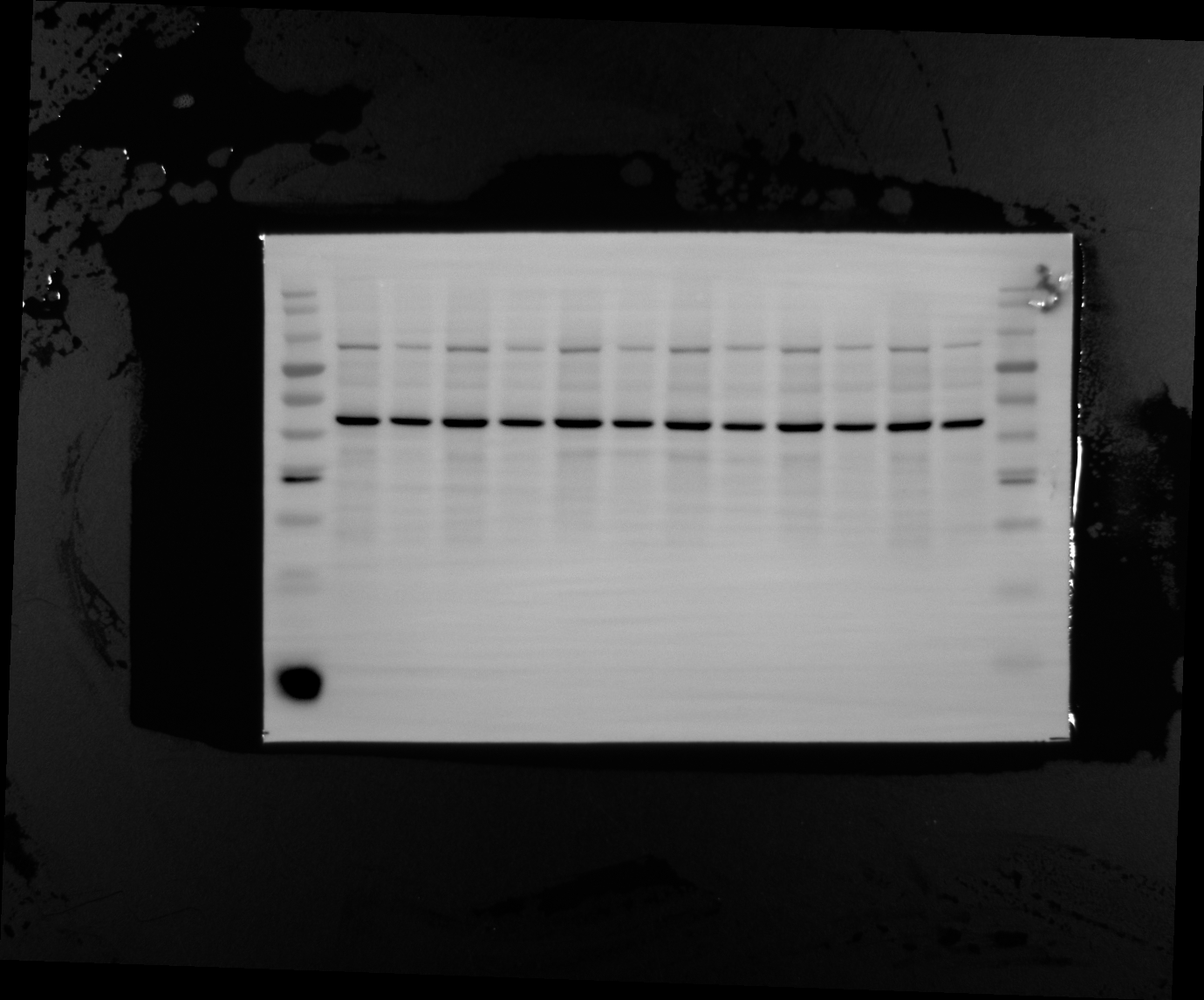


α-tubulin-Figure5


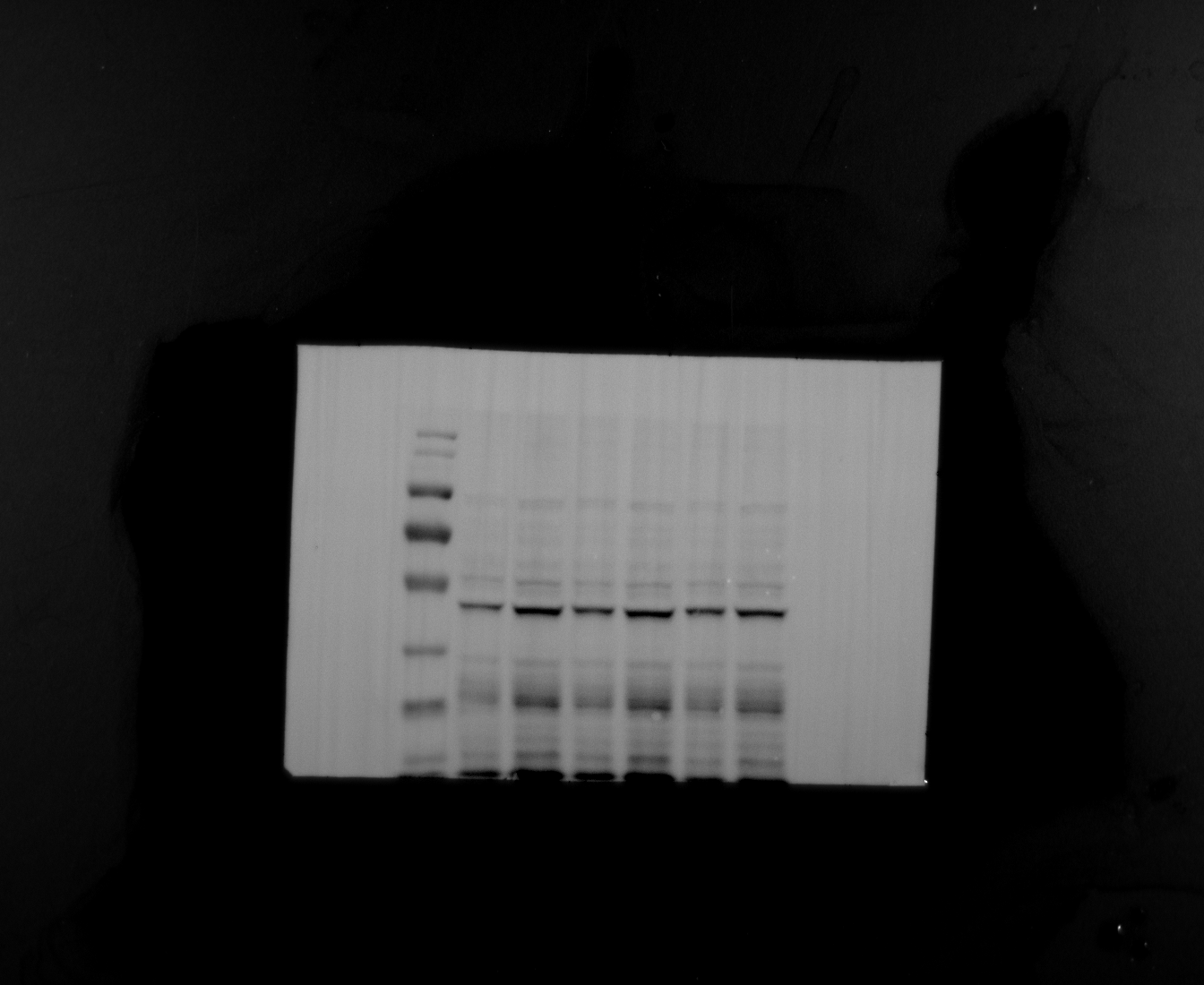


CCNL1-Figure7


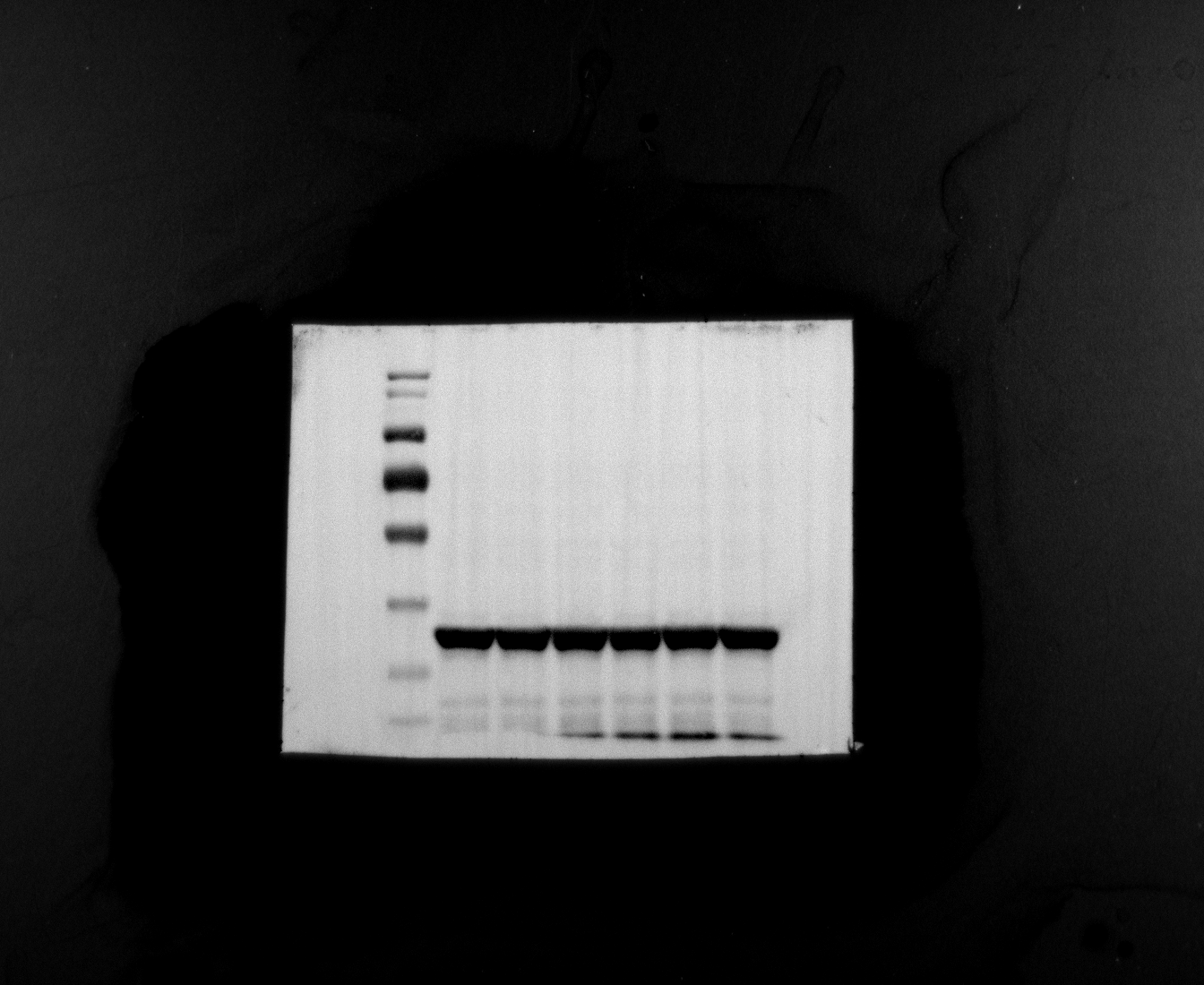


GAPDH-Figure7


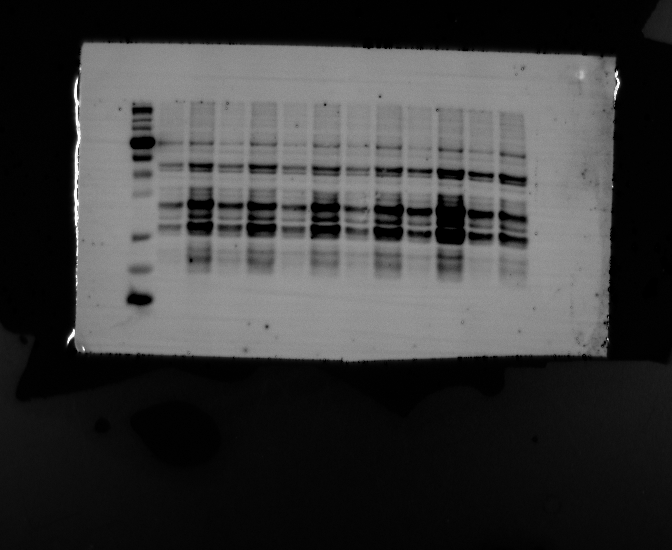


TCF4-Figure8


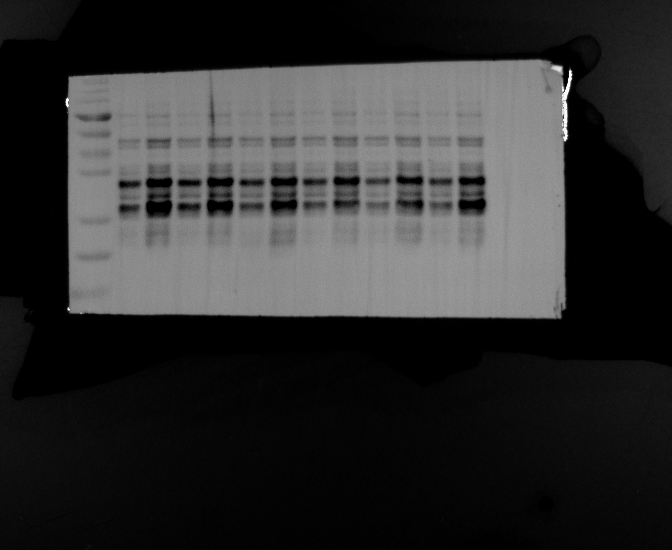


p-GSK-3β-Figure8


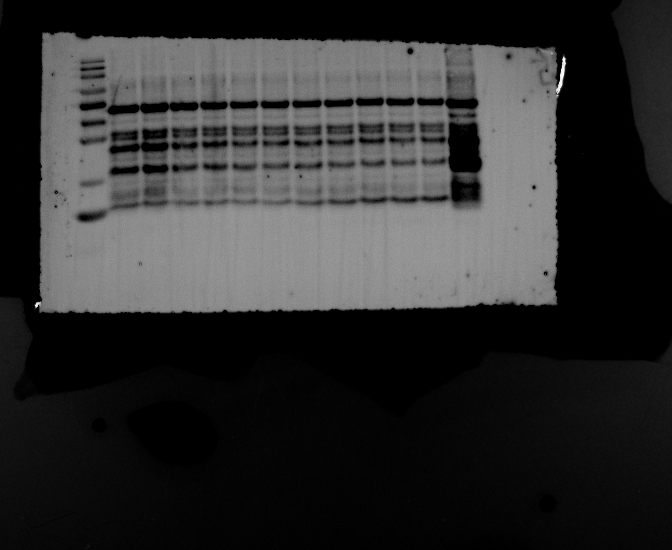


GSK-3β-Figure8


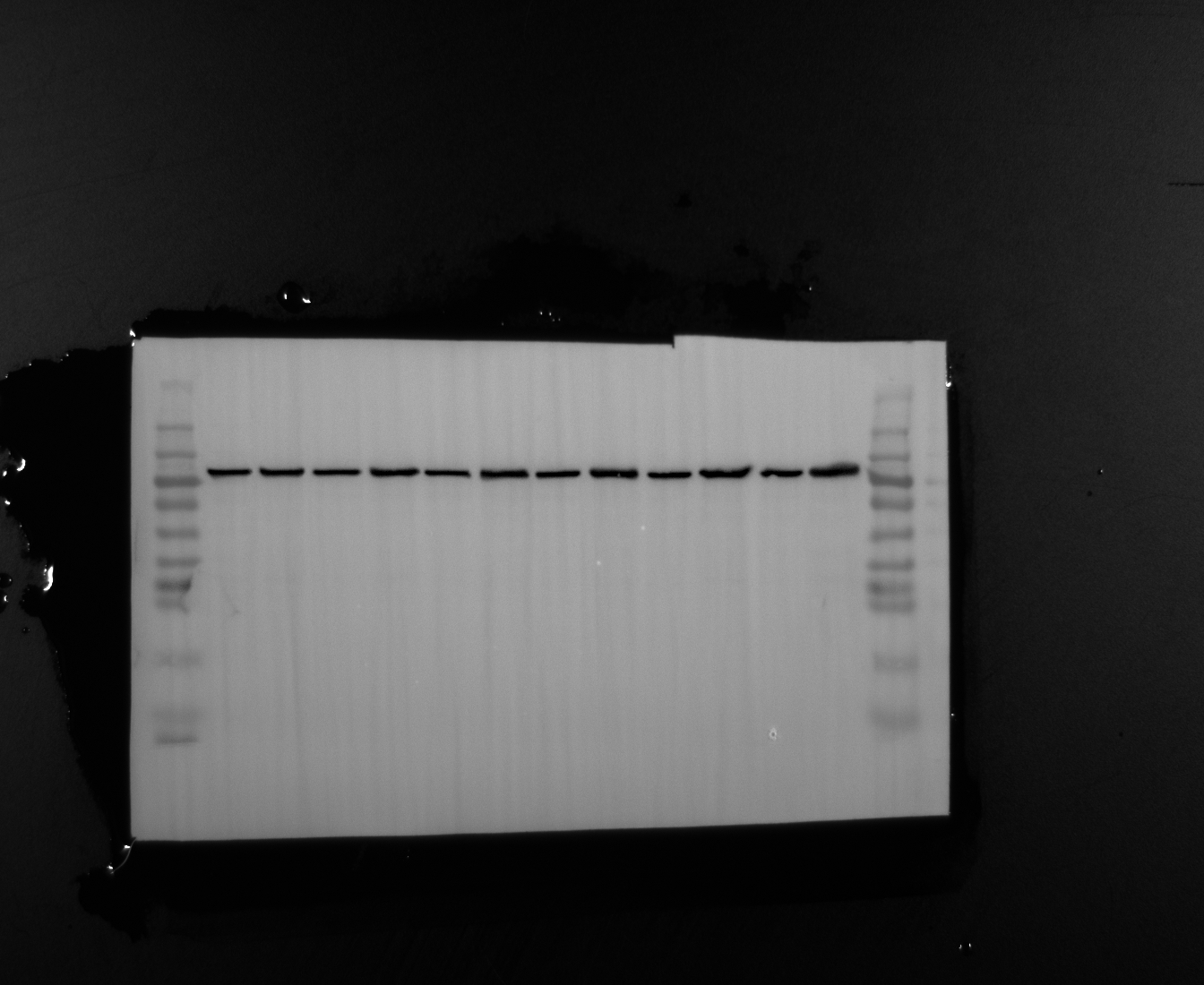


β-catenin-Figure8


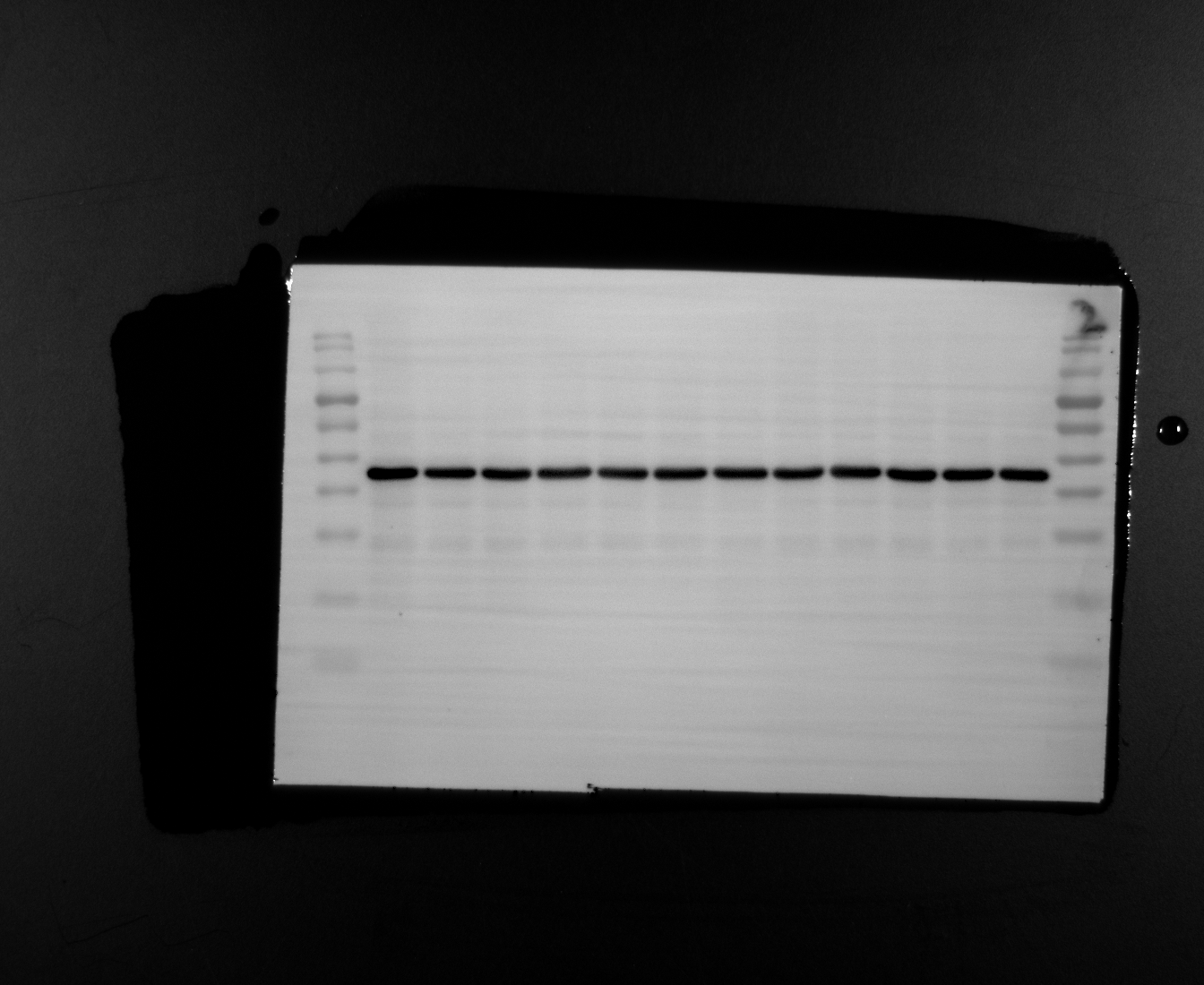


GAPDH-Figure8
