# Supplementary material for: Tumor-derived exosomal miR-199b-5p promotes proliferation and epithelial-mesenchymal transition in non-small cell lung cancer by targeting CCNL1
Source: Transl Oncol. 2025 Oct 16;62:102564. doi: 10.1016/j.tranon.2025.102564 (PMC12554135; doi:10.1016/j.tranon.2025.102564)
Supplement: Supplementary file 2 [file mmc2.docx]

**Extraction of exosome by ultracentrifugation method**

Serum from healthy volunteers and NSCLC patients were melted at 37 ℃, then moved the sample to a new centrifuge tube, centrifuge at 2000 × g, 4℃ for 30 min. The supernatant was carefully transferred to a new centrifuge tube, centrifuged again at 10000 × g, 4 ℃, and 45 min. The supernatant was collected and filtered through a 0.45 μm membrane, then collect the filtered solution. The filtered liquid was then transfer to a new centrifuge tube, selected an overspeed rotor, centrifuge at 4 ℃ and 100000 × g for 70 min. The supernatant was removed, and resuspended in 10 mL of pre cooled 1 × PBS, the previous centrifuge step was repeated. The supernatant was removed, and resuspend in 150 μL of pre cooled 1 × PBS, 20 μL used for electron microscopy, 10 μL for particle size, 10-100 μL for protein extracted, and the remaining exosome were stored at -80 ℃ for the following .

**Transmission electron microscopy**

Exosome samples (10 μL) were added onto a copper mesh to precipitate for 1 min, and removed the floating liquid with filter paper. Uranyl acetate (10 μL) were dropwise onto a copper mesh to precipitate for 1 min, then washed five times with distilled water and dried with filter paper at room temperature. Transmission electron microscopy (HT-7700, Hitachi, Japan) was performed to obtain image at 100 kV.

**Nanoparticle tracking analysis**

Exosome size distribution and particle concentration was measured using NanoFCM (N30E, Xiamen, China), software version NTA2.3 using the following setting; detection level 7, camera level 14, detection time: 5 × 30 s.

**MiR-199b-5p mimic and lentiviruses plasmids**

The miRNA mimic (miR-199b-5p mimic) and negative control miRNA mimic were purchased from Tsingke Biotech Co., Ltd. (Beijing, China). Lipofectamine 3000 transfection reagent (Invitrogen, USA) was used to transfect miRNAs. MiR-199b-5p mimic or negative control mimic was transfected cells with 3 μg. After transfection for 48 h, collectd cells for the following experiments. QRT-PCR was used to evaluate the transfection efficiency.

Lentiviruses expressing miR-199b-5p negative control (NC) or miR-199b-5p (miR-199b-5p UP) were obtained from Tsingke Biotech Co., Ltd. (Beijing, China). Lentiviruses plasmids were constructed based on pLVX-shRNA2-Luc-EGFP-Puro vectors. Cells were transfected with the indicated plasmid according to manufacturer’s protocol for Lipofectamine 3000 (Invitrogen, USA) 24 h prior to treatment.
